# RNA granules: functional compartments or incidental condensates?

**DOI:** 10.1101/gad.350518.123

**Published:** 2023-05-01

**Authors:** Andrea Putnam, Laura Thomas, Geraldine Seydoux

**Affiliations:** Howard Hughes Medical Institute, Department of Molecular Biology and Genetics, Johns Hopkins University, Baltimore, Maryland 21205, USA

**Keywords:** phase separation, RNA granules, ribonucleoprotein complexes, condensates

## Abstract

RNA granules are mesoscale assemblies that form in the absence of limiting membranes. RNA granules contain factors for RNA biogenesis and turnover and are often assumed to represent specialized compartments for RNA biochemistry. Recent evidence suggests that RNA granules assemble by phase separation of subsoluble ribonucleoprotein (RNP) complexes that partially demix from the cytoplasm or nucleoplasm. We explore the possibility that some RNA granules are nonessential condensation by-products that arise when RNP complexes exceed their solubility limit as a consequence of cellular activity, stress, or aging. We describe the use of evolutionary and mutational analyses and single-molecule techniques to distinguish functional RNA granules from “incidental condensates.”

## Abstract (precis)

In this review, Putnam et al. tackle our current understanding of RNA granules, intracellular phase-separated sites of RNA metabolism that contain factors for RNA biogenesis and turnover and are often assumed to represent specialized compartments for RNA biochemistry. The authors discuss the possibility that some RNA granules are condensation by-products that form when subsoluble RNP complexes saturate the cytoplasm or nucleoplasm, and introduce the term “incidental condensates” to refer to condensates that are tolerated by cells but do not add functionality beyond that provided by the soluble pool of saturating RNP complexes. Finally, they consider best practices for distinguishing functional RNA granules from incidental condensates.

RNA granules are intracellular RNA–protein assemblies not enclosed by membranes that range in size from ∼100 nm to several micrometers. RNA granules have been observed in animal, fungus, plant, and prokaryotic cells and fall into three general classes: ubiquitous, cell type-specific, and stress-induced. Over 20 types of RNA granules have been described so far, each with a unique composition, comprising in some cases dozens of proteins and thousands of RNAs (Emenecker et al. 2020; Gao et al. 2021; Lacroix and Audas 2022; Hirose et al. 2023; Rostam et al. 2023).

Proteins enriched in RNA granules function in many aspects of RNA metabolism, from transcription and processing in the nucleus to translation and RNA turnover in the cytoplasm. By extension, RNA granules are often assumed to represent functional compartments that house RNA-focused activities that require the granule environment (Hirose et al. 2023). For example, nucleoli assemble around nascent ribosomal RNAs and concentrate ribosomal proteins and ribosome assembly factors, implicating the nucleolus as the main cellular compartment for ribosome biogenesis (Lafontaine et al. 2021). Similarly, many other RNA granules have been assigned putative functions based on composition, including P-bodies as sites of mRNA storage or decay and nuclear speckles as sites of mRNA splicing (Standart and Weil 2018; Faber et al. 2022; Vidya and Duchaine 2022).

Recent findings have linked RNA granule assembly to phase separation of RNA–protein (RNP) complexes (Shin and Brangwynne 2017). Phase separation is a thermodynamic process that causes interacting molecules to “demix” from the cytoplasm or nucleoplasm into dense condensates (Hyman et al. 2014). Unlike compartments delimited by membranes, which require energy to assemble and maintain, condensates form spontaneously under conditions such as high concentration, when components exceed their solubility limit. In this review, we explore the possibility that some RNA granules are condensation by-products that form when subsoluble RNP complexes saturate the cytoplasm or nucleoplasm. We introduce the term “incidental condensates” to refer to condensates that are tolerated by cells but do not add functionality beyond that provided by the soluble pool of saturating RNP complexes. We begin by describing how the biophysical properties of phase-separated condensates provide a strong theoretical framework to describe the dynamics and composition of RNA granules and, at the same time, raise questions as to their potential role as cellular compartments. Next, we review experimental evidence in support of and against functions commonly assigned to RNA granules. Finally, we consider best practices for distinguishing functional RNA granules from incidental condensates. Table 1 summarizes the main themes addressed in this review.

**Table 1.**
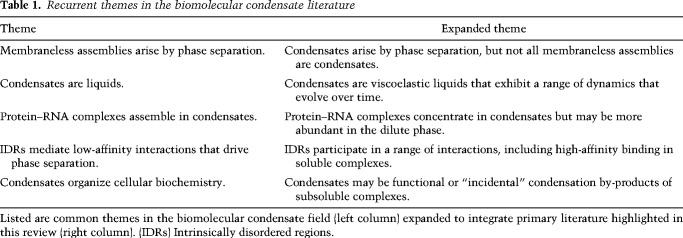
Recurrent themes in the biomolecular condensate literature

## Properties of condensates

### What is a condensate?

**Figure 1.**
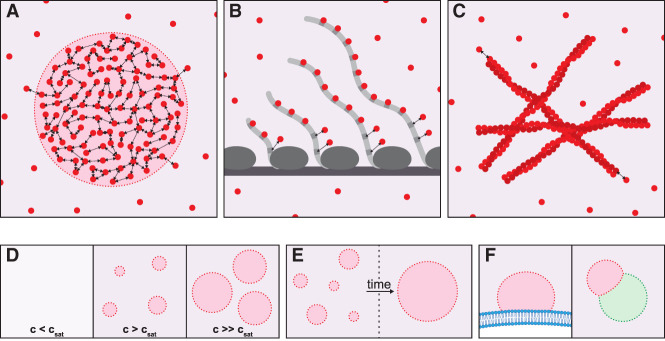
Condensates and other assemblies. (*A*) Condensates arise when diffusive multivalent molecules (red circles) interact reversibly (double arrows) to form a dynamic network. Condensates are defined by an interface (red dotted line), with associated surface tension separating the condensed phase (red) from the dilute phase (purple). The surface tension arises from the energy differential between molecules at the interface (which are pulled into the condensate by their neighbors) and molecules in the interior. The molecules inside the condensate experience a chemical and diffusive environment distinct from the dilute phase. (*B*) A multivalent scaffold (such as nascent RNA molecules) can concentrate proteins (red) that bind to the scaffold (gray). Such an assembly may resemble a condensate by microscopy but does not possess an interface and therefore is not phase-separated. However, this type of assembly could evolve into a condensate if the proteins, in addition to binding to the scaffold, also interact with one another and binding to the scaffold causes the proteins to exceed *c*_sat_ locally. (*C*) Proteins containing low-complexity, prion-like domains can interact via β-sheet stacking to form extended fibers in multiple dimensions. This type of assembly does not constitute a phase-separated condensate but could arise within a condensate that concentrates proteins with prion-like domains. (*D*–*F*) Properties of condensates. (*D*) Condensates form above *c*_sat_ (saturation concentration), the maximum concentration allowed in the dilute phase. Above *c*_sat_, further increases in concentration cause the condensates to grow larger without any changes to the concentration in the dilute phase, which remains at *c*_sat_. However, this theoretical prediction is difficult to apply in vivo, where multiple components contribute to *c*_sat_, leading to complex concentration-dependent behaviors (Riback et al. 2020). (*E*) Surface tension drives condensates to minimize surface area, causing them to coarsen over time to create fewer, larger condensates with lower surface:volume ratios. The time scale of coarsening will depend on the material properties of the condensates (less dynamic condensates will coarsen more slowly). Also, agents that adsorb to the interface can reduce surface tension and coarsening. (*F*) Condensates wet surfaces, including membranes (blue) and other condensate types (green) that provide favorable interaction interfaces (Gouveia et al. 2022). For example, P granules wet nuclear membranes, and P-bodies wet stress granules.

Banani et al. (2017) first introduced the term “biomolecular condensate” to refer to any mesoscale assembly that concentrates biomolecules without the help of a limiting membrane, irrespective of mechanism. In this review, we use the term “condensate” in a narrower sense to refer to the product of condensation or, more precisely, phase separation (Shin and Brangwynne 2017). Phase separation is an emergent property of diffusive molecules that interact with each other in solution. Above a critical concentration (*c*_sat_), the sum of favorable intermolecular interactions offsets the entropic cost of demixing, causing interacting molecules to redistribute (“phase separate”) into a dense phase (condensate) and a dilute phase (Hyman et al. 2014). Unlike ordered assemblies, where molecules assume specific configurations (e.g., actin in microfilaments), molecules in condensates adopt multiple conformations and binding stoichiometries, exchanging binding partners within the condensate and exchanging with the dilute phase. The time scales of these dynamics can vary greatly, causing the condensates to appear “liquid-like” or “solid-like,” although most biological condensates are likely neither simple liquids nor solids but rather viscoelastic fluids (Jawerth et al. 2020; Mittag and Pappu 2022). Condensates do not have a prescribed size and theoretically can grow infinitely if provided unlimited components. Although molecules in the condensate flux in and out, condensates have a sharp inside/outside boundary or interface (Fig. 1A). Molecules inside the condensates experience a chemical and diffusive environment distinct from that experienced by molecules in the surrounding “dilute” phase (cytoplasm or nucleoplasm) (Israelachvili 2011).

It is important to emphasize that not all assemblies visible by microscopy are necessarily phase-separated condensates (McSwiggen et al. 2019b). Reversible binding to a multivalent scaffold, such as DNA or RNA molecules, can colocalize diffusive proteins into dynamic assemblies that superficially resemble condensates (Fig. 1B). Amyloid protein aggregation—and possibly other forms of polymerization where proteins form ordered fibrils that extend in three dimensions—could also in principle generate supramolecular assemblies that resemble condensates (Fig. 1C; Kato et al. 2022). Unlike condensates, however, these types of assemblies do not have a distinct “surface” (interface) separating molecules in the assembly from the surrounding medium and thus do not create true “compartments” (Fig. 1).

In principle, condensates can be distinguished from other assemblies by their unique growth, fusion, and wetting properties, specified by their interfacial and internal dynamics. For example, condensates exhibit switch-like growth and degrowth in response to changes in concentration, fuse upon contact, and wet surfaces such as membranes (Fig. 1D–F), as illustrated by Brangwynne et al. (2009), who demonstrated that the P granules of *Caenorhabditis elegans* are liquid-like condensates. However, these types of analyses are difficult to perform on assemblies that have slow dynamics and/or are diffraction-limited in size. Other reviews have addressed the challenges associated with determining whether RNA granules correspond to condensates, other assembly types, or a mixture of the two (Erdel and Rippe 2018; Alberti et al. 2019; McSwiggen et al. 2019b; Forman-Kay et al. 2022). In the following sections, we describe how the biophysical properties of condensates explain certain aspects of RNA granule dynamics in cells and, at the same time, complicate function determination.

### Phase separation is a spontaneous, concentration-dependent process

Phase separation is driven by associative (“binding”) interactions and segregative (“repulsive”) interactions that cause molecules to sort into distinct phases above *c*_sat_. The landmark study of Li et al. (2012) showed that sequence-specific protein-binding domains can drive phase separation when present in multiple copies (multivalency) capable of generating large networks of interacting molecules. Long, flexible RNA molecules are ideal multivalent scaffolds for RNA-binding proteins and essential components of several types of RNA granules (Decker et al. 2022). Condensation is also enhanced by sequence-nonspecific interactions, involving protein–protein, protein–RNA, and possibly also RNA–RNA interactions (Box 1).

Box 1. **What types of interactions drive the formation of RNA granules?**

Genetic studies have shown that sequence-specific protein-binding domains contribute to RNA granule assembly in cells. For example, oligomerization domains in Edc3, G3BP, and PGL-1 are required to assemble P-bodies, stress granules, and P granules, respectively, likely because these domains mediate the formation of subsoluble RNP complexes (Ling et al. 2008; Hanazawa et al. 2011; Kedersha et al. 2016; Guillén-Boixet et al. 2020; Sanders et al. 2020; Yang et al. 2020). In addition to globular domains, RNA-binding proteins often also contain intrinsically disordered regions (IDRs) that can phase-separate in isolation in vitro. Best studied is the FUS IDR, which is stabilized in condensates by a variety of binding interactions involving most amino acids along the length of the IDR, which remains disordered in the condensates (Murthy et al. 2019; Martin and Holehouse 2020; Peran and Mittag 2020). Whether IDR–IDR interactions drive the phase separation of native RNA granules in cells, however, is less clear. IDRs are rarely sufficient to drive phase separation in cells (unless overexpressed) but can augment the condensation of oligomerizing globular domains by linking condensation to environmental inputs such as RNA availability, pH, oxidation state, and temperature (Riback et al. 2017; Franzmann et al. 2018; Kato et al. 2019; Guillén-Boixet et al. 2020; Iserman et al. 2020; Sanders et al. 2020; Yang et al. 2020; Putnam and Seydoux 2023). For example, the IDR of the P granule protein MEG-3 is not essential for condensation but binds RNA in a sequence-nonspecific manner and is required to recruit low-translation mRNAs to P granules (Lee et al. 2020; Schmidt et al. 2021)

RNA can promote or disrupt protein condensates. In vitro, RNA can lower the *c*_sat_ of RNA-binding proteins by functioning as a multivalent scaffold or raise *c*_sat_ by competing with protein–protein interactions (Zhang et al. 2015; Saha et al. 2016; Maharana et al. 2018; Lee et al. 2020; Rhine et al. 2020). Both effects have been observed in cells. Some nuclear proteins, such as nucleolar RNA-binding proteins, condense around point sources of nascent RNAs, likely because binding to colocalized RNAs raises their concentration locally above *c*_sat_, allowing them to phase-separate (Berry et al. 2015; Lawrimore et al. 2021). Other RNA-binding proteins, such as FUS, require RNA binding and high RNA concentrations to remain soluble in nuclei (Maharana et al. 2018). In the cytoplasm, the assembly of RNA granules is often stimulated by abundant low-translation transcripts. For example, P-bodies assemble around low-translation deadenylated mRNAs, and P granules and stress granules assemble around low-translation, polyadenylated mRNAs (Tharun and Parker 2001; Hubstenberger et al. 2017; Khong et al. 2017; Lee et al. 2020).

RNA molecules can phase-separate in vitro even in the absence of proteins through non-sequence-specific π–π, hydrogen-bonding, and electrostatic interactions (Nakano et al. 2007; Aumiller et al. 2016; Van Treeck et al. 2018; Onuchic et al. 2019; Bevilacqua et al. 2022; Forman-Kay et al. 2022). These observations have led to the proposal that RNA–RNA interactions contribute to condensation in cells, especially under stress conditions that block translation initiation and release thousands of “naked” mRNAs in the cytoplasm (Van Treeck et al. 2018). mRNAs sort into homotypic clusters inside RNA granules in *Drosophila* embryos (Niepielko et al. 2018; Trcek et al. 2020), and RNA structure influences the material properties of RNA– protein condensates in vitro and in reconstituted systems in tissue culture (Maharana et al. 2018; Ma et al. 2021; Roden and Gladfelter 2021; Decker et al. 2022). Remarkably, some RNAs appear immobile in condensates even when bound by dynamic proteins (Moon et al. 2019; Cabral et al. 2022), indicating that RNAs can assemble static scaffolds inside RNA granules. In summary, RNA molecules have a high propensity for condensation, especially when not engaged in translation, and can play a dominant role in specifying condensate organization and dynamics.

**Figure 2.**
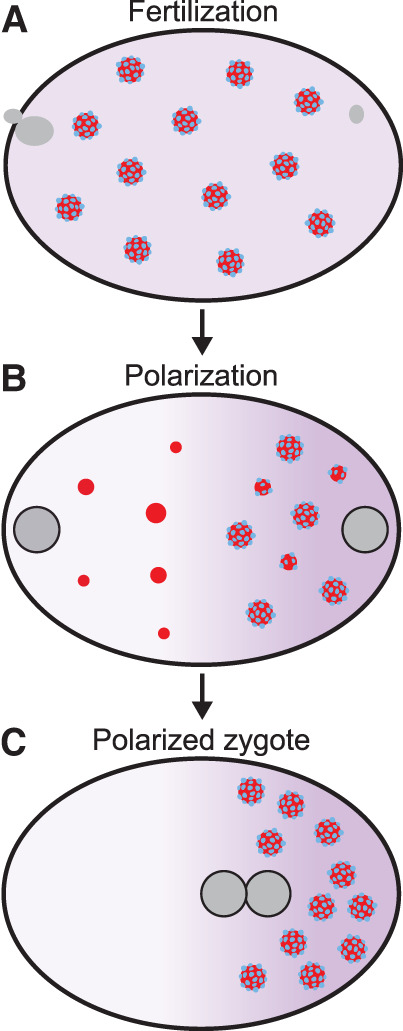
P granules are condensates that undergo localized dissolution and condensation in the *C. elegans* zygote. P granules consist of a central liquid core (containing dozens of proteins; red) covered by solid-like clusters (blue) adsorbed to the interface of the liquid core. All P granule components exchange between the granules and cytoplasm. The solid clusters recruit the kinase DYRK3, which accelerates P granule/cytoplasm exchange. The solid clusters also lower surface tension to stabilize P granules against coarsening (“Pickering effect”). (*A*) In unpolarized zygotes, P granules distribute throughout the uniformly saturated cytoplasm. (*B*) During polarization, P granules dissolve in the anterior cytoplasm and grow in the posterior cytoplasm in response to two spatial inputs: (1) A subset of P granule components enrich in the posterior, forming a saturation gradient across the cytoplasm, and (2) interfacial clusters are depleted from anterior granules and enriched on posterior granules by an unknown mechanism, preferentially stabilizing posterior granules. (*C*) In polarized zygotes, P granules are only found in the supersaturated environment of the posterior cytoplasm.

Because phase separation is a concentration-dependent equilibrium process, no energy input is required to initiate condensation. Changes in the concentration, valency, or binding affinity of protein and/or RNA molecules are sufficient to induce condensation (or dissolution). Consistent with these theoretical predictions, RNA granule assembly in cells has been correlated with changes in the concentration or valency of proteins and RNAs. For example, the polarized condensation and dissolution of P granules coincides temporarily with the formation of concentration gradients across the cytoplasm of the *C. elegans* zygote (Fig. 2; Brangwynne et al. 2009; Wang et al. 2014; Saha et al. 2016; Smith et al. 2016; Folkmann et al. 2021). The assembly of stress granules and P-bodies is linked to translation inhibition and RNA deadenylation, respectively, which increase the pool of RNA molecules available for binding by stress granule and P-body proteins (Chen and Shyu 2013; Bounedjah et al. 2014). Some nuclear condensates, such as nucleoli, condense around point sources of nascent RNAs (Berry et al. 2015; Lawrimore et al. 2021). Condensation is also predicted to be affected by factors that impact the solvation capacity of the cytoplasm or nucleoplasm. Lowering ribosome numbers in yeast and HEK293 cells decreased the condensation of an artificial condensate, which could be rescued partially by osmotic shock, likely due to changes in molecular crowding (Delarue et al. 2018). Although no energy is required to initiate condensation, cells use ATP-consuming mechanisms to counter condensation and enhance the solubility of proteins and RNAs (Box 2).

Box 2. **Cells expend energy to minimize condensation and maintain condensates in a dynamic state**

Theoretical considerations indicate that multivalent polymers in initially liquid condensates will “harden” over time by maximizing interactions with their neighbors, leading to “kinetic trapping,” where molecules are unable to exit or enter the condensate (Ranganathan and Shakhnovich 2020; Chatterjee et al. 2022; Jiang et al. 2023). Experiments in vitro have shown that proteins in RNA granules form condensates that either (1) remain liquid for hours and evolve glass-like properties over days (Jawerth et al. 2020), (2) start out liquid and arrest dynamics within minutes (Bose et al. 2022), or (3) are immediately solid within the experimental time frame (Putnam et al. 2019; Jawerth et al. 2020; Bose et al. 2022). Cryo-EM analyses have confirmed that initially liquid condensates aged in vitro maintain an amorphous appearance with no internal structure, consistent with an arrested liquid (Jawerth et al. 2020; Bose et al. 2022).

Condensate hardening is potentially problematic because slow dynamics extend the time scale at which condensates will respond to environmental perturbations. When placed in a dilute environment below *c*_sat_, condensates will dissolve (lose molecules) at a rate proportional to the rate at which molecules in the condensates liberate themselves from their neighbors to enter the dilute phase. Similarly, when placed in a concentrated environment above *c*_sat_, condensates will grow on a time scale proportional to the rate at which molecules at the interface rearrange to accommodate new neighbors incoming from the dilute phase. These theoretical considerations generally mean that reducing binding interactions between condensate components will increase *c*_sat_, increase the rate of disassembly in undersaturated conditions (*c* < *c*_sat_), and increase the rate of growth in oversaturated conditions (*c* > *c*_sat_).

Several lines of evidence suggest that cells expend energy to enhance the solubility of RNAs and proteins and maintain condensates in a responsive, dynamic state. The cytoplasm of *Escherichia coli* behaves like a glass-forming liquid that requires constant energetic input from metabolism to remain fluid, becoming “vitrified” under low energy conditions (Parry et al. 2014). Similarly, the liquid-like dynamics of nucleoli require ATP (Brangwynne et al. 2011). Several ATP-dependent mechanisms have been identified that minimize condensation in cells. ATP-consuming protein chaperones clear heat-induced condensates (Yoo et al. 2022). DEAD-box (DDX) proteins are a large family of ATPases proposed to tune condensation by binding RNA (Hilliker et al. 2011; Elbaum-Garfinkle et al. 2015; Mugler et al. 2016; Hondele et al. 2019; Marnik et al. 2019). DDX proteins bind RNA when ATP-bound and release RNA upon ATP hydrolysis (Putnam and Jankowsky 2013). DDX proteins that contain intrinsically disordered regions promote condensation when bound to RNA and trigger dissolution upon ATP hydrolysis (Mugler et al. 2016; Hondele et al. 2019). The DDX translation initiation factor eIF4A dissolves RNA condensates in vitro in an ATP-dependent manner and limits stress granules assembly in cells (Tauber et al. 2020). Protein modifications can also modulate solubility. Nucleoporins stockpiled in oocytes depend on phosphorylation and sugar modifications to remain soluble and limit the formation of potentially toxic condensates (Thomas et al. 2022). Phosphorylation by the DYRK kinase MBK-2 is essential to accelerate P granule dynamics and ensure that their polarized dissolution and condensation occur sufficiently fast to keep up with embryonic cell divisions (Folkmann et al. 2021). The prion-like domain of FUS exhibits strong selection for phosphorylation sites predicted to prevent hardening (Dasmeh and Wagner 2021). The emerging view is that many cellular components are naturally close to saturation and cells have evolved energy-consuming mechanisms to limit condensation. A corollary is that treatments that interfere with energy production will lead to condensation.

### The ‘dark’ side of condensation: the dilute phase

A common assumption is that molecules in RNA granules are highly concentrated in the granules and only active in the granule environment. This assumption has led to the widespread hypothesis that RNA granules represent functional compartments, akin to organelles, that house specialized functions not possible in the more dilute environment of the cytoplasm or nucleoplasm (Banani et al. 2017; Fare et al. 2021). However, the realization that many RNA granules likely are condensates that arise by phase separation challenges this view. As described above, phase separation involves partitioning of molecules between two phases: a dilute phase and a condensed phase. The fraction of molecules in each phase will depend on the partition coefficient and on concentration. At concentrations right above *c*_sat_, the highest concentration permitted in the dilute phase, only a small fraction of molecules will populate the condensates. If molecules in the dilute phase are also active (i.e., in RNP complexes), redistribution of activity from the dilute phase to the condensates will be minimal.

Several lines of evidence indicate that phase-separating proteins also assemble complexes in the dilute phase. First, classical biochemistry experiments have defined many protein and protein–RNA complexes that assemble in solution (Musacchio 2022). Binding domains defined by those experiments drive phase separation when multimerized in vitro (e.g., Li et al. 2012) and are required to assemble RNA granules in vivo (Ling et al. 2008; Hanazawa et al. 2011; Kedersha et al. 2016; Guillén-Boixet et al. 2020; Sanders et al. 2020; Yang et al. 2020). These observations suggest that phase separation in cells is intimately linked to the networking potential of multivalent macromolecular complexes, as recently articulated by Mittag and Pappu (2022). Consistent with this view, proteomic analyses in cell lysates have revealed that the connectivity of stress granule proteins does not change following stress granule assembly, suggesting that the RNP complexes that populate stress granules also exist as soluble species in the cytoplasm (Markmiller et al. 2018; Youn et al. 2018). Similarly, certain yeast mutants that lack P-bodies still assemble P-body protein complexes that can be detected by nanoparticle tracking (Rao and Parker 2017). Interestingly, even simple model condensate proteins that self-interact using distributed IDR–IDR interactions (e.g., FUS) form heterogeneous oligomers or “clusters” in solution (Murthy et al. 2019; Zhao et al. 2021; Kar et al. 2022; Seim et al. 2022). The clusters range from a handful to hundreds of molecules, grow larger with increasing concentrations, and are thought to lower *c*_sat_ by increasing valency and decreasing solubility as a function of size (Kar et al. 2022). The polyQ condensing protein Whi3 of *Ashbya gossypii* also forms soluble oligomers in vitro, although in that context the soluble oligomers appear to compete with condensation (Seim et al. 2022). The emerging view is that RNA granules arise from condensation of RNP complexes that also form in the dilute phase.

How many RNP complexes are left in the dilute phase when condensates form in cells? Quantitative studies addressing this question for native RNA granules are rare (Lyon et al. 2021). A systematic survey in yeast revealed that most P-body proteins are present in higher proportions in the cytoplasm. When P-body proteins are labeled with GFP, P-bodies appear brighter than the cytoplasm—but the total fluorescence intensity in the cytoplasm is actually higher than that in P-bodies—due to modest enrichment in P-bodies and the small fraction of total cell volume occupied by P-bodies (Xing et al. 2020). Similarly, most mRNAs recruited to stress granules in mammalian cells or P granules in *C. elegans* are more abundant in the cytoplasm (Khong et al. 2017; Moon et al. 2019; Lee et al. 2020; Glauninger et al. 2022). Although the dilute phase may appear “dark,” it may be the primary compartment for mRNA regulation.

### Incidental condensates

**Figure 3.**
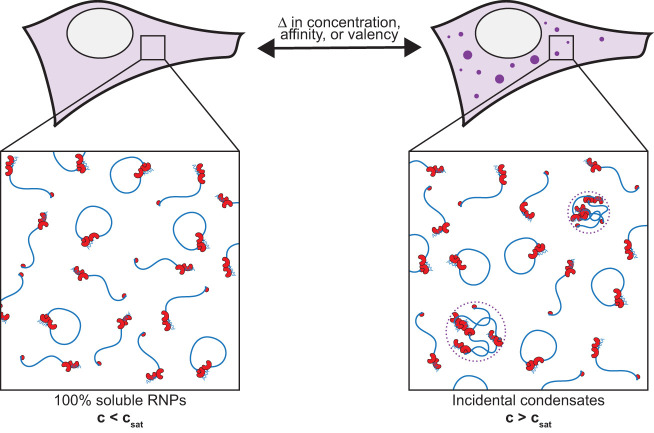
Near saturation conditions, changes in concentration, valency, or affinity of RNP complexes (or in the solvation capacity of the cytoplasm or nucleoplasm) are sufficient to induce condensation or dissolution of RNP complexes. Incidental condensates appear concentrated when visualized by fluorescence microscopy but contain only a fraction of RNP complexes, many of which remain in the dilute phase. Incidental condensates are tolerated by cells but add no functionality beyond that provided by RNP complexes in the dilute phase. Although nonessential, incidental condensates can be useful markers of cellular activity supported by saturating complexes, as well as markers of stress and aging (see the text).

Based on the considerations above, we propose a new null hypothesis for RNA granule assembly in cells that does not impose functionality: RNA granules arise when RNP complexes exceed their solubility limit in the cytoplasm (or nucleoplasm) and a fraction demixes into phase-separated condensates. If the condensates enrich active RNP complexes or change their activity, the condensates will represent functional compartments that house (or suppress) the activity associated with the RNPs localized therein. On the other hand, if the condensates do not create (or localize) new activity, the condensates will have no functional consequences. We refer to such nonfunctional condensates as “incidental” to denote the fact that their assembly is a secondary consequence of developmental, physiological, or stress-induced changes in the concentration, affinity, or valency of RNP complexes, leading to oversaturation of the cytoplasm or nucleoplasm (Fig. 3). In the next section, we examine proposed functions for RNA granules in light of this new null hypothesis.

## Possible functions for RNA granules

RNA granules typically have been assigned functions based on composition and/or biochemical experiments using condensates reconstituted in vitro (Lyon et al. 2021). Six general themes have emerged, which we consider in turn, evaluating supporting evidence and alternative interpretations. Exemplary RNA granules and their proposed functions are listed in Table 2.

**Table 2.**
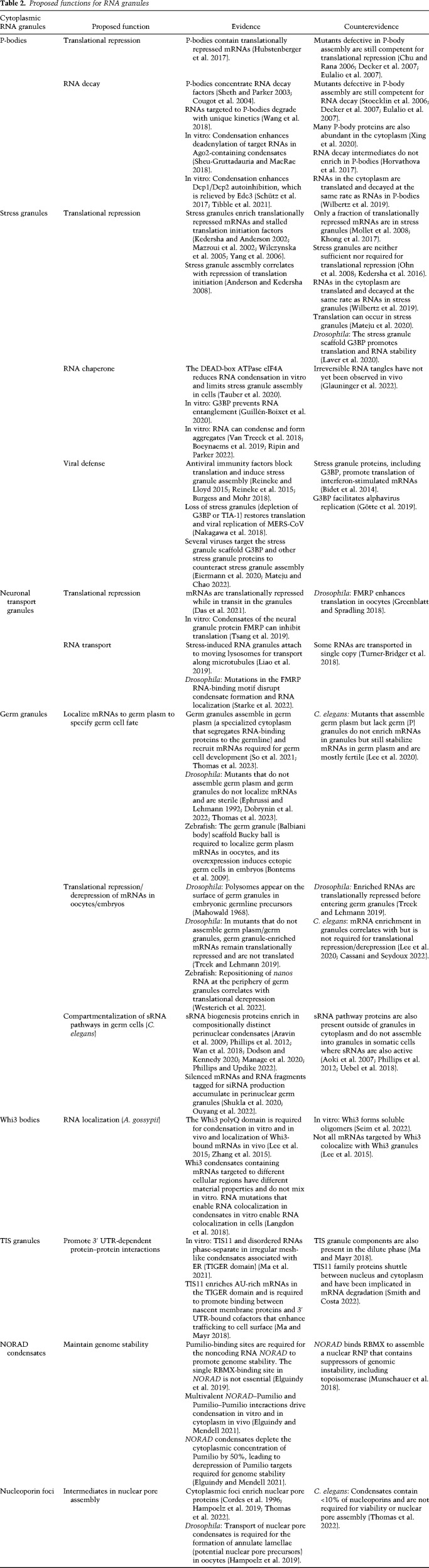
Proposed functions for RNA granules

### Translational repression

An often-cited role for RNA granules is translational repression. RNA granules have been proposed to block the translation of mRNAs in granules by excluding ribosomes and/or enriching factors that compete with the translational machinery for access to transcripts (Parker et al. 2022). Observations in several granule models, however, argue that assembly into RNA granules is a consequence rather than a cause of translation repression. First, targeting of mRNAs to P-bodies by miRNAs, or to stress granules in stressed cells, correlates with translational repression, but translational repression does not require P-bodies or stress granules (Eulalio et al. 2007; Ohn et al. 2008; Kedersha et al. 2016). Second, RNAs that enrich in the polar granules of *Drosophila* oocytes are translationally repressed prior to entering the granules. Third, mRNAs that enrich in P granules maintain low ribosome occupancy and translational repression even in embryos that lack P granules (Lee et al. 2020).

The weight of the evidence today favors a model in which loss of elongating ribosomes enhances RNA condensation into stress granules, P-bodies, and P granules by generating “naked” RNA molecules that can interact with each other and with RNA-binding proteins. Indeed, treatments that decrease translation initiation and lead to ribosome runoff increase recruitment of RNAs into RNA granules. In contrast, active or stalled elongating ribosomes limit the assembly of stress granules, P-bodies, and P granules and the accumulation of translated mRNAs in stress granules and P granules (Mazroui et al. 2002; Brengues et al. 2005; Teixeira et al. 2005; Khong and Parker 2018; Lee et al. 2020). Elongating ribosomes, however, are not an absolute barrier to accumulation in stress granules, as translating mRNAs have been observed on the surface and inside stress granules (Mateju et al. 2020). These findings do not exclude the possibility that phase separation of translational repressors enhances their activity, as suggested for FMRP (Kim et al. 2019b; Tsang et al. 2019), but this hypothesis remains to be tested directly in vivo.

### RNA storage and suppression of RNA entanglement

Another proposed function for RNA granules is long-term storage of translationally repressed mRNAs that eventually recycle back into the cytoplasm when favorable conditions return. When yeast cells are shifted from stress conditions to conditions favoring growth, transcripts in P-bodies are returned to translation (Brengues et al. 2005; Bhattacharyya et al. 2006). Similarly, RNAs and proteins in stress granules are released into the cytoplasm and “recycled” following release from stress (Wallace et al. 2015; Wilbertz et al. 2019; Das et al. 2022). In *C. elegans*, mRNAs in P granules disperse back into the cytoplasm upon translational activation in the germline founder cell (Lee et al. 2020; Parker et al. 2020; Cassani and Seydoux 2022). RNA granules are also prominent in oocytes and early embryos, which stockpile maternal mRNAs to be used for embryonic development (So et al. 2021). Embryonic RNA granules in zebrafish accumulate mRNAs during oogenesis that become activated for translation in developing embryos (Sato et al. 2022). Ripin and Parker (2022) have proposed that packaging of RNAs into RNA granules could limit the tendency of “naked” RNAs to become hopelessly tangled by cohousing RNA with RNA-binding proteins that compete with RNA–RNA interactions. Consistent with this possibility, stress granules contain proteins that can prevent RNA entanglement and melt RNA-only condensates in vitro (Sauer et al. 2019; Guillén-Boixet et al. 2020; Tauber et al. 2020; He et al. 2021).

However, direct evidence for RNA entanglement in cells remains lacking. After recovery from stress, RNAs in the cytoplasm are translated and decayed at the same rate as RNAs in stress granules and P-bodies (Wilbertz et al. 2019). Similarly, in mutants defective in P granule assembly, translational activation in the germline founder cell proceeds with the same timing as in wild type (Lee et al. 2020; Cassani and Seydoux 2022). These observations suggest that storage in RNA granules is not essential to maintain the functionality of translationally repressed mRNAs, but more precise measurements may be needed to reveal a possible quality control role for RNA granules.

### RNA transport, enrichment, and localized ‘translation factories’

One of the best-documented roles for RNA granules is to localize RNAs to specific cellular areas by active transport or passive enrichment. RNA granules in neurons are an example of active transport: Neuronal granules travel down axonal processes, transporting mRNAs to synapses and other sites distal from the cell body (Das et al. 2019). One proposed mechanism involves “hitchhiking” using a specific tether that links the granules to lysosomes that travel on microtubules (Liao et al. 2019). Other RNA granules localize mRNAs by assembling at specific cellular locations and passively trapping mRNAs. In *Drosophila*, mRNAs coding for germ cell determinants are captured as single molecules by germ granules that assemble at the posterior-most end of oocytes, marking the site where germ cells will form in the future embryo (Niepielko et al. 2018). Similarly, in *C. elegans* embryos, repeated cycles of polarized assembly and disassembly of P granules in synchrony with cell division enrich maternal mRNAs in the germline founder cell (Schmidt et al. 2021). In the multinucleate hyphae of *A. gossypii*, the phase-separating RNA-binding protein Whi3 is required to both regulate translation and localize transcripts to specific cellular areas (Lee et al. 2015; Zhang et al. 2015), although whether the latter involves transport or local entrapment is not known.

mRNAs in granules are often translationally repressed during transport and activated postlocalization. In *Drosophila*, mRNAs in germ granules are translated at specific developmental stages, possibly by polysomes on the surface of the granules (Mahowald 1968; Rangan et al. 2009). In *C. elegans*, P granule mRNAs are released into the cytoplasm coincident with translation, although in this system, granule localization is not a prerequisite for translational activation (Lee et al. 2020; Schmidt et al. 2021; Cassani and Seydoux 2022). Other RNA granules have been proposed to function as localized “translation factories” that coordinate the translation of mRNAs coding for proteins that function in the same process. For example, granules that enrich glycolytic enzymes also contain mRNAs coding for those same enzymes (Morales-Polanco et al. 2021). Similarly, growing yeast enrich mRNAs coding for translation factors at bud tips—sites of rapid growth (Pizzinga et al. 2019). mRNAs coding for axonemal dynein concentrate in a granule at the growing end of cilia in *Drosophila* sperm (Fingerhut and Yamashita 2020). In some cases, the RNAs become localized in granules indirectly through cotranslational protein targeting, as suggested for RNAs that colocalize with centrosomes, the cell cortex, and nucleoporin condensates (Sepulveda et al. 2018; Hampoelz et al. 2019; Parker et al. 2020). In *Drosophila* oocytes, nucleoporin condensates have been proposed to function as preassembly sites for nuclear pore complexes stored in membrane structures called annulate lamellae (Hampoelz et al. 2019). A study in *C. elegans*, however, suggests that nucleoporin condensates are dispensable for viability and correspond to minor condensation by-products of a large stockpile of highly cohesive FG-Nups maintained near *c*_sat_ in oocytes for use later during embryogenesis (Thomas et al. 2022). Whether RNAs localize to condensates to promote, or as a consequence of, binding between encoded proteins during translation remains to be determined.

### Compartmentalization of biochemical reactions

By enriching and excluding specific components, RNA granules could also theoretically function to compartmentalize biochemical reactions in cells, separating reaction substrates from products for example. Early pioneering studies revealed that condensates assembled with the intrinsically disordered region of the helicase Ddx4 exclude duplex nucleic acids, enrich single strands, and even melt duplex structures, acting as “biomolecular filters” (Nott et al. 2015, 2016). Based on in vitro reconstitutions and observations in *Xenopus* oocytes that assemble nucleoli around extrachromosomal rDNA, the nucleolus has been modeled as a multilayered condensate where differential binding to nascent versus folded ribosomal RNA promotes vectorial transport of assembly intermediates through the nucleolar layers (Feric et al. 2016; Mitrea et al. 2016; Yao et al. 2019; Riback et al. 2020; Lafontaine et al. 2021). Consistent with vectorial flow, cryo-electron tomography studies suggest that ribosomal intermediates distribute in a gradient in the outer layer of the nucleolus, with fully mature complexes enriching at the periphery (Erdmann et al. 2021). However, interpretation of the functional significance of nucleolar layers is complicated by the observations that ribosome assembly factors cycle between active and latent forms, nucleolar morphology varies considerably between cells and species, and other functions have been attributed to nucleoli besides ribosome production (Hernandez-Verdun et al. 2010; Tartakoff et al. 2022; Hori et al. 2023). Tagging of a subset of rDNA repeats in human cells revealed that rRNAs remain around their site of origin while in nucleoli, suggesting that ribosome biogenesis occurs entirely in subnucleolar territories anchored around the transcriptionally active rDNA repeats (Mangan and McStay 2021). Consistent with this view, a recent study reported that disruption of nucleolar layers is compatible with viability and fertility under normal growth conditions in *C. elegans* (Spaulding et al. 2022). One hypothesis posits that the protein-rich nucleolar layers arise as a consequence of ribosome biogenesis proximal to rDNA repeats and function primarily as storage sites for latent ribosome biogenesis factors and partially unfolded proteins during heat stress (Alberti and Carra 2019; Frottin et al. 2019; Tartakoff et al. 2022).

The model of the nucleolus as a compartmentalized condensate that supports RNA transcription and processing has been extended to mRNA-coding genes (Hnisz et al. 2017). The C-terminal domain of RNA polymerase II, as well as several IDR-containing transcription factors, undergoes phase separation in vitro. Clusters of RNA polymerase II and transcription factors have been observed around active transcription sites in nuclei, although whether these represent phase-separated condensates or smaller assemblies (“hubs”) is under debate (McSwiggen et al. 2019a; Darzacq and Tjian 2022; Palacio and Taatjes 2022; Rippe and Papantonis 2022). Interestingly, for several natural and engineered transcription factors, propensity for phase separation correlates with transcriptional output, and condensates nucleated by the IDR of Mediator are sufficient to activate transcription of a reporter gene (Wei et al. 2020; Trojanowski et al. 2022; Lyons et al. 2023). However, one study examining accumulation of nascent RNAs in real time found no correlation between transcriptional output and the presence of a condensate at the locus (Trojanowski et al. 2022), and another found that overexpression of an IDR driving condensation inhibits gene expression (Chong et al. 2022). Rather than promoting condensation, IDRs could facilitate the assembly of soluble complexes linking transcription factors to RNA polymerase II (Ferrie et al. 2022; Rippe and Papantonis 2022). IDRs can facilitate complex assembly by dynamic binding to ordered domains or to other IDRs, in some cases with remarkably high (picomolar) affinity (Pontius 1993; Borgia et al. 2018; Hong et al. 2020). Because the same domains that drive condensation could also drive the formation of smaller, non-phase-separated assemblies, assigning a function to condensates is challenging. One possibility is that both condensates and non-phase-separated assemblies facilitate transcription but at different loci (Palacio and Taatjes 2022).

RNA-rich condensates that form in the cytoplasm have also been proposed to compartmentalize RNA-based processes. For example, in the *C. elegans* adult germline, components of the small RNA biosynthetic machinery sort into at least four distinct perinuclear germ granules, each with a unique composition (Sundby et al. 2021). However, this complex organization is unlikely to be essential for small RNA biogenesis, since small RNAs are also active in somatic cells, which do not assemble condensates (Phillips et al. 2012). Compartmentalization of small RNA biogenesis in germ cells has been proposed to prevent dangerous feed-forward silencing loops that could silence genes for generations, but direct evidence for this hypothesis is not yet available (Ouyang and Seydoux 2022). Alternatively, multiphase condensation could simply be a consequence of the different types of RNPs involved in small RNA biogenesis and their relatively high concentration in the adult germline, a tissue with above-average rates of transcription. Consistent with this view, treatments that lower transcription reduce germ granule condensation (Sheth et al. 2010).

RNA granules have also been suggested to promote the assembly of protein complexes on nascent proteins during translation. TIS granules are ER-associated condensates that enrich transcripts coding for trafficked proteins. The condensate-forming TIS11B protein recruits mRNAs to TIS granules and facilitates their binding to SET, a cofactor that promotes protein trafficking to the plasma membrane (Ma and Mayr 2018). How TIS granules enhance such interactions is not yet known, but transient entrapment of newly translated proteins in the dense environment of the TIS11B condensates has been proposed as a possible mechanism (Chen and Mayr 2022).

### Enhanced RNA biochemistry

An exciting possibility is that condensation of RNA in protein-rich condensates creates a solvent environment uniquely suited for RNA biochemistry. Studies using artificial dextran-rich condensates found them to be potent RNA concentrators and enhancers of ribozyme activity (Strulson et al. 2012; Poudyal et al. 2019). Similarly, condensates of the Argonaute Ago2 and the GW182 protein TNRC6B sequester Argonaute-bound RNAs and accelerate their deadenylation in vitro (Sheu-Gruttadauria and MacRae 2018). Reconstitution of minimal P-bodies using purified components has revealed that condensation can modulate RNA-decapping activity (Schütz et al. 2017; Tibble et al. 2021). Condensation stabilizes the decapping complex Dcp1/Dcp2 in an autoinhibited conformation while preserving the complex's ability to respond to stimulation by the decapping activator Edc3. The lower basal activity of Dcp1/Dcp2 in condensates causes Edc3 to raise decapping activity by 90-fold in condensates, compared with a mere threefold in solution (Tibble et al. 2021). Together, these studies demonstrate that, in principle, condensates can support RNP enzymology and enhance reactions by increasing concentrations and modulating protein and RNA conformation. Direct evidence that native RNA granules provide a unique solvent environment for RNA biochemistry in cells, however, is still lacking.

### Titration of soluble RNPs

Several lines of evidence suggest that some RNA granules affect RNA biochemistry indirectly by removing RNA regulators from the soluble pool. Nuclear speckles and paraspeckles accumulate splicing and other nuclear proteins, which when released in the nucleoplasm lead to dysregulated gene expression (Hirose et al. 2014; Hochberg-Laufer et al. 2019). Condensation of poly(A)-binding protein is enhanced by heat stress and antagonized by RNA, driving poly(A)-binding protein into RNA-free gel-like condensates that enhance stress resistance (Riback et al. 2017). In human cells, the noncoding RNA *NORAD* is induced by DNA damage and condenses the normally soluble translational repressor Pumilio. High *NORAD* copy number and valency (18 Pumilio-binding sites) drive highly efficient condensation of Pumilio, depleting the soluble pool by half, which in turn activates the translation of Pumilio mRNA targets (Elguindy and Mendell 2021). These findings demonstrate that efficient condensation can tune RNA biochemistry by reducing the concentration of RNA regulators in the nucleoplasm or cytoplasm.

## Best practices for determining RNA granule function

Several challenges complicate the assessment of RNA granule function in cells. First, because most RNA granules likely assemble by phase separation of RNP complexes also present in the dilute phase, it is not straightforward to parse out whether the activity under study comes from complexes in the dilute phase, the condensates, or both. Second, “part lists” alone cannot be used to predict function, since some proteins may become inactive or change function in the condensate environment. Third, condensates may facilitate activities that only become essential under specific conditions, such as protein and/or RNA chaperoning under stress, and these functions may require new assays to fully assess. Last, because condensation is a spontaneous process sensitive to fluctuations in concentration and binding affinities, the possibility that some condensates are not functional and incidental to the assembly of subsoluble complexes cannot be discounted. In vitro reconstitutions will continue to serve as powerful tools to explore the effect of condensation on enzymatic activity and protein and RNA folding (Lyon et al. 2021). In the next sections, we consider in vivo experiments that can be used to complement in vitro reconstitutions to probe the function of native RNA granules.

### Quantitative analyses of protein and RNA enrichment in condensates

A first step toward functional characterization of an RNA granule is to determine the percentage of molecules inside versus outside of the granules. These experiments should be performed using probes that detect endogenous components, without transgenes that could lead to overexpression, and ideally in living animals to minimize nonphysiological stresses associated with cell culture. Care should also be taken to avoid fixation or microscopy conditions that enhance or suppress condensation (Uebel and Phillips 2019; Elaswad et al. 2022a; Irgen-Gioro et al. 2022). The relatively small volume of RNA granules compared with the rest of the cell may mean that, even when prominent by microscopy, RNA granules may only account for a small proportion of molecules, as demonstrated for stress granules, P-bodies, and nucleoporin condensates (Khong et al. 2017; Xing et al. 2020; Thomas et al. 2022). Such a finding may help rule out models that require a significant proportion of RNPs to localize to the condensates (such as inhibitory/titration models) but does not necessarily rule out other functions. For example, in situ hybridization experiments revealed that P granules capture at most only ∼30% of molecules for a specific translationally repressed transcript, ruling out granule localization as causal for translational repression (Lee et al. 2020). Comparisons of embryos with and without P granules, however, confirmed that recruitment into granules correlates with enrichment of those transcripts in the germline founder cell, supporting a role for P granules in RNA localization (Schmidt et al. 2021).

### Titration experiments

Because condensation is ultrasensitive to concentration, one approach to determine whether condensates are essential is to titrate the concentration of a critical condensate scaffold. The titration range should flank *c*_sat_ to generate cells with and without condensates and should be small enough to not significantly affect the concentration of soluble complexes. This approach has been used to evaluate the functional relevance of transcriptional condensates (Chong et al. 2022; Trojanowski et al. 2022). For example, by monitoring transcriptional output in real time in cells expressing variable levels of a condensing transcription factor, the investigators concluded that the presence of condensate on the transcribing locus had no, or a slightly negative, effect on transcriptional output (Trojanowski et al. 2022). However, one drawback to this approach is that, in the case of multiscaffold condensates, titration of only one scaffold could skew condensate composition, leading to nonfunctional condensates (Riback et al. 2020).

### Genetic and evolutionary analyses

Another approach is to use genetic mutants to correlate activity in cells and in reconstituted systems. Mutations that affect condensation without affecting enzymatic activity or RNA binding can be used to disentangle effects due to loss or disruption of condensates versus loss of RNP complex activity (Riback et al. 2017; Iserman et al. 2020; Tibble et al. 2021). In such analyses, it is important to verify that the mutations do not affect RNP complex assembly in the dilute phase. Sequences between binding sites (“spacer” sequences) can be good mutagenic targets if they contribute to the overall solubility of RNP complexes, but their effects may be weak due to redundancy (Kar et al. 2022; Mittag and Pappu 2022). Evolutionary analyses can be used to reveal conserved sequence features selected to tune condensation to environmental inputs (Pritišanac et al. 2020). For example, sequence analyses comparing 351 poly(A)-binding protein orthologs revealed a strong selection signature for hydrophobic amino acids in a proline-rich low-complexity domain. These observations guided the construction of an allelic series with predicted reduced hydrophobicity, which was then used to demonstrate a correlation between propensity for phase separation in vitro and stress resistance in vivo (Riback et al. 2017). Similarly, examination of orthologs of the *Saccharomyces cerevisiae* Ded1 RNA helicase revealed a region required for temperature-induced condensation. Mutations that lowered the temperature threshold required for Ded1 condensation were detrimental to yeast fitness and revealed that Ded1 condensation is adapted to species-specific thermal niches (Iserman et al. 2020). Interestingly, in both cases, condensation was correlated with loss of activity, allowing for a selective shift in the types of mRNAs translated in the cytoplasm upon heat-induced stress. These examples also illustrate the importance of assaying function under a range of conditions that explore the full fitness landscape, as some condensates may only contribute to organismal fitness under stress or other specific conditions.

### In vivo imaging

Mutational analyses can be complemented with imaging to directly locate activity in live cells. In cases where the RNA granules are predicted to promote RNA biochemistry, it may be necessary to develop probes to measure enzymatic activity in situ. As mentioned above, studies examining transcriptional output in real time have shown that the presence of a condensate at a transcriptionally active locus does not always correlate with increased transcriptional activity (Trojanowski et al. 2022). Single-molecule studies have also debunked assumptions about stress granules as compartments incompatible with translation by showing that RNAs inside the granules are accessible to the translation machinery (Mateju et al. 2020). Single-molecule analyses also revealed that mRNA degradation events occur throughout the cytoplasm and do not enrich in P-bodies (Horvathova et al. 2017). Recent advances in superresolution microscopy techniques that permit precise counting of molecules colocalized in cells may also provide insights into whether condensates or smaller clusters (or both) underlie biological processes (Bond et al. 2022; Castells-Garcia et al. 2022).

Quantitative observations in cells can also be used to refine in vitro reconstitutions. Molecular parameters measured in vivo (concentrations, diffusion rates/internal viscosity, and surface tension) can be used to adjust conditions for reconstitutions in vitro to better match the in vivo setting. Parameters measured in vivo and in vitro in turn can inform theory to test and refine quantitative models (e.g., Folkmann et al. 2021). Ultimately, combinations of in vivo, in vitro, and in silico experiments will be needed to develop a quantitative understanding of RNA granule function and dynamics.

## Outlook—incidental condensates as markers of cellular activity, stress, and aging

Phase separation of multivalent RNP complexes into condensates is an attractive model for the compartmentalization of RNA-focused activities in cells. For some native RNA granules, observations in cells are consistent with the physical properties of condensates, providing a sound theoretical framework for modeling RNA granule dynamics. Because the inside of phase-separated condensates is chemically and diffusionally distinct from the surrounding phase, condensates hold great promise as compartments with unique biochemistry. At the same time, the exquisite sensitivity of phase separation to small changes in solubility and concentration raises the possibility that some condensates are “incidental,” tolerated by cells as by-products of cellular activity but providing no new function.

Although not functional, incidental condensates could still be useful to experimentalists as reporters of cellular activities involving subsoluble RNP complexes. For example, the sudden appearance of P-bodies signals the onset of maternal mRNA turnover in embryos (Gallo et al. 2008). Analysis of the composition and dynamics of incidental condensates could report on the types of molecular complexes and their dynamics in the cytoplasm or nucleoplasm during cellular and developmental transitions. Mutations that disrupt incidental condensates could be used to guide the identification of binding sites in proteins and/or RNAs that mediate RNP complex assembly in vivo. Incidental condensates could also serve as useful markers of stress responses and aging, as exemplified by stress granules that arise under stress conditions that block translation initiation (Kedersha and Anderson 2002; Kedersha et al. 2005) and cytoplasmic nucleoporin condensates, whose assembly is enhanced by heat shock and organismal aging (Patterson et al. 2011; Thomas et al. 2022). Because cells use energy-consuming mechanisms to counter condensation (Box 2), incidental condensates may also prove useful markers to identify quiescent cells with lower ATP production. For example, arrested oocytes accumulate many condensate types (Jud et al. 2007; Elaswad et al. 2022b), which we speculate arise as a consequence of the suppression of metabolic activity that accompanies dormancy (Rodríguez-Nuevo et al. 2022). Incidental condensates are tolerated by healthy cells but could in theory become toxic if allowed to evolve into irreversible sinks that deplete soluble proteins and RNAs, as suggested by several studies linking accelerated condensation to disease (Boeynaems et al. 2018; Nedelsky and Taylor 2022). Understanding how cells minimize incidental condensates may suggest strategies to reverse pathological condensates and aggregates. Finally, we anticipate that in vivo experiments, combined with in vitro reconstitutions conducted under physiological conditions, will help distinguish incidental condensates from functional RNA granules where evolution has harnessed phase separation to drive biological function.

## References

[GAD350518PUTC1] Alberti S, Carra S. 2019. Nucleolus: a liquid droplet compartment for misbehaving proteins. Curr Biol 29: R930–R932. 10.1016/j.cub.2019.08.013 31593669

[GAD350518PUTC2] Alberti S, Gladfelter A, Mittag T. 2019. Considerations and challenges in studying liquid–liquid phase separation and biomolecular condensates. Cell 176: 419–434. 10.1016/j.cell.2018.12.035 30682370 PMC6445271

[GAD350518PUTC3] Anderson P, Kedersha N. 2008. Stress granules: the Tao of RNA triage. Trends Biochem Sci 33: 141–150. 10.1016/j.tibs.2007.12.003 18291657

[GAD350518PUTC4] Aoki K, Moriguchi H, Yoshioka T, Okawa K, Tabara H. 2007. In vitro analyses of the production and activity of secondary small interfering RNAs in *C. elegans*. EMBO J 26: 5007–5019. 10.1038/sj.emboj.7601910 18007599 PMC2140100

[GAD350518PUTC5] Aravin AA, van der Heijden GW, Castañeda J, Vagin VV, Hannon GJ, Bortvin A. 2009. Cytoplasmic compartmentalization of the fetal piRNA pathway in mice. PLoS Genet 5: e1000764. 10.1371/journal.pgen.1000764 20011505 PMC2785470

[GAD350518PUTC6] Aumiller WM Jr, Pir Cakmak F, Davis BW, Keating CD. 2016. RNA-based coacervates as a model for membraneless organelles: formation, properties, and interfacial liposome assembly. Langmuir 32: 10042–10053. 10.1021/acs.langmuir.6b02499 27599198

[GAD350518PUTC7] Banani SF, Lee HO, Hyman AA, Rosen MK. 2017. Biomolecular condensates: organizers of cellular biochemistry. Nat Rev Mol Cell Biol 18: 285–298. 10.1038/nrm.2017.7 28225081 PMC7434221

[GAD350518PUTC8] Berry J, Weber SC, Vaidya N, Haataja M, Brangwynne CP. 2015. RNA transcription modulates phase transition-driven nuclear body assembly. Proc Natl Acad Sci 112: E5237–E5245. 10.1073/pnas.1509317112 26351690 PMC4586886

[GAD350518PUTC9] Bevilacqua PC, Williams AM, Chou HL, Assmann SM. 2022. RNA multimerization as an organizing force for liquid–liquid phase separation. RNA 28: 16–26. 10.1261/rna.078999.121 34706977 PMC8675289

[GAD350518PUTC10] Bhattacharyya SN, Habermacher R, Martine U, Closs EI, Filipowicz W. 2006. Relief of microRNA-mediated translational repression in human cells subjected to stress. Cell 125: 1111–1124. 10.1016/j.cell.2006.04.031 16777601

[GAD350518PUTC11] Bidet K, Dadlani D, Garcia-Blanco MA. 2014. G3BP1, G3BP2 and CAPRIN1 are required for translation of interferon stimulated mRNAs and are targeted by a dengue virus non-coding RNA. PLoS Pathog 10: e1004242. 10.1371/journal.ppat.1004242 24992036 PMC4081823

[GAD350518PUTC12] Boeynaems S, Alberti S, Fawzi NL, Mittag T, Polymenidou M, Rousseau F, Schymkowitz J, Shorter J, Wolozin B, Van Den Bosch L, 2018. Protein phase separation: a new phase in cell biology. Trends Cell Biol 28: 420–435. 10.1016/j.tcb.2018.02.004 29602697 PMC6034118

[GAD350518PUTC13] Boeynaems S, Holehouse AS, Weinhardt V, Kovacs D, Van Lindt J, Larabell C, Van Den Bosch L, Das R, Tompa PS, Pappu RV, 2019. Spontaneous driving forces give rise to protein–RNA condensates with coexisting phases and complex material properties. Proc Natl Acad Sci 116: 7889–7898. 10.1073/pnas.1821038116 30926670 PMC6475405

[GAD350518PUTC14] Boija A, Klein IA, Sabari BR, Dall'Agnese A, Coffey EL, Zamudio AV, Li CH, Shrinivas K, Manteiga JC, Hannett NM, 2018. Transcription factors activate genes through the phase-separation capacity of their activation domains. Cell 175: 1842–1855.e16. 10.1016/j.cell.2018.10.042 30449618 PMC6295254

[GAD350518PUTC15] Bond C, Santiago-Ruiz AN, Tang Q, Lakadamyali M. 2022. Technological advances in super-resolution microscopy to study cellular processes. Mol Cell 82: 315–332. 10.1016/j.molcel.2021.12.022 35063099 PMC8852216

[GAD350518PUTC16] Bontems F, Stein A, Marlow F, Lyautey J, Gupta T, Mullins MC, Dosch R. 2009. Bucky ball organizes germ plasm assembly in zebrafish. Curr Biol 19: 414–422. 10.1016/j.cub.2009.01.038 19249209

[GAD350518PUTC17] Borgia A, Borgia MB, Bugge K, Kissling VM, Heidarsson PO, Fernandes CB, Sottini A, Soranno A, Buholzer KJ, Nettels D, 2018. Extreme disorder in an ultrahigh-affinity protein complex. Nature 555: 61–66. 10.1038/nature25762 29466338 PMC6264893

[GAD350518PUTC18] Bose M, Lampe M, Mahamid J, Ephrussi A. 2022. Liquid-to-solid phase transition of oskar ribonucleoprotein granules is essential for their function in *Drosophila* embryonic development. Cell 185: 1308–1324.e23. 10.1016/j.cell.2022.02.022 35325593 PMC9042795

[GAD350518PUTC19] Bounedjah O, Desforges B, Wu T-D, Pioche-Durieu C, Marco S, Hamon L, Curmi PA, Guerquin-Kern J-L, Piétrement O, Pastré D. 2014. Free mRNA in excess upon polysome dissociation is a scaffold for protein multimerization to form stress granules. Nucleic Acids Res 42: 8678–8691. 10.1093/nar/gku582 25013173 PMC4117795

[GAD350518PUTC20] Brangwynne CP, Eckmann CR, Courson DS, Rybarska A, Hoege C, Gharakhani J, Jülicher F, Hyman AA. 2009. Germline P granules are liquid droplets that localize by controlled dissolution/condensation. Science 324: 1729–1732. 10.1126/science.1172046 19460965

[GAD350518PUTC21] Brangwynne CP, Mitchison TJ, Hyman AA. 2011. Active liquid-like behavior of nucleoli determines their size and shape in *Xenopus laevis* oocytes. Proc Natl Acad Sci 108: 4334–4339. 10.1073/pnas.1017150108 21368180 PMC3060270

[GAD350518PUTC22] Brengues M, Teixeira D, Parker R. 2005. Movement of eukaryotic mRNAs between polysomes and cytoplasmic processing bodies. Science 310: 486–489. 10.1126/science.1115791 16141371 PMC1863069

[GAD350518PUTC23] Burgess HM, Mohr I. 2018. Defining the role of stress granules in innate immune suppression by the herpes simplex virus 1 endoribonuclease VHS. J Virol 92: e00829-18. 10.1128/JVI.00829-18 29793959 PMC6052315

[GAD350518PUTC24] Cabral SE, Otis JP, Mowry KL. 2022. Multivalent interactions with RNA drive recruitment and dynamics in biomolecular condensates in *Xenopus* oocytes. iScience 25: 104811. 10.1016/j.isci.2022.104811 35982794 PMC9379569

[GAD350518PUTC25] Cassani M, Seydoux G. 2022. Specialized germline P-bodies are required to specify germ cell fate in *Caenorhabditis elegans* embryos. Development 149: dev200920. 10.1242/dev.200920 36196602 PMC9686995

[GAD350518PUTC26] Castells-Garcia A, Ed-daoui I, González-Almela E, Vicario C, Ottestrom J, Lakadamyali M, Neguembor MV, Cosma MP. 2022. Super resolution microscopy reveals how elongating RNA polymerase II and nascent RNA interact with nucleosome clutches. Nucleic Acids Res 50: 175–190. 10.1093/nar/gkab1215 34929735 PMC8754629

[GAD350518PUTC27] Chatterjee S, Kan Y, Brzezinski M, Koynov K, Regy RM, Murthy AC, Burke KA, Michels JJ, Mittal J, Fawzi NL, 2022. Reversible kinetic trapping of FUS biomolecular condensates. Adv Sci (Weinh) 9: 2104247. 10.1002/advs.202104247 34862761 PMC8811844

[GAD350518PUTC28] Chen L-L, Carmichael GG. 2009. Altered nuclear retention of mRNAs containing inverted repeats in human embryonic stem cells: functional role of a nuclear noncoding RNA. Mol Cell 35: 467–478. 10.1016/j.molcel.2009.06.027 19716791 PMC2749223

[GAD350518PUTC29] Chen X, Mayr C. 2022. A working model for condensate RNA-binding proteins as matchmakers for protein complex assembly. RNA 28: 76–87. 10.1261/rna.078995.121 34706978 PMC8675283

[GAD350518PUTC30] Chen C-YA, Shyu A-B. 2013. Deadenylation and P-bodies. Adv Exp Med Biol 768: 183–195. 10.1007/978-1-4614-5107-5_11 23224971 PMC3804309

[GAD350518PUTC31] Cho W-K, Spille J-H, Hecht M, Lee C, Li C, Grube V, Cisse II. 2018. Mediator and RNA polymerase II clusters associate in transcription-dependent condensates. Science 361: 412–415. 10.1126/science.aar4199 29930094 PMC6543815

[GAD350518PUTC32] Chong S, Graham TGW, Dugast-Darzacq C, Dailey GM, Darzacq X, Tjian R. 2022. Tuning levels of low-complexity domain interactions to modulate endogenous oncogenic transcription. Mol Cell 82: 2084–2097.e5. 10.1016/j.molcel.2022.04.007 35483357

[GAD350518PUTC33] Chu C, Rana TM. 2006. Translation repression in human cells by microRNA-induced gene silencing requires RCK/p54. PLoS Biol 4: e210. 10.1371/journal.pbio.0040210 16756390 PMC1475773

[GAD350518PUTC34] Cordes VC, Reidenbach S, Franke WW. 1996. Cytoplasmic annulate lamellae in cultured cells: composition, distribution, and mitotic behavior. Cell Tissue Res 284: 177–191. 10.1007/s004410050578 8625385

[GAD350518PUTC35] Cougot N, Babajko S, Séraphin B. 2004. Cytoplasmic foci are sites of mRNA decay in human cells. J Cell Biol 165: 31–40. 10.1083/jcb.200309008 15067023 PMC2172085

[GAD350518PUTC36] Courchaine E, Gelles-Watnick S, Machyna M, Straube K, Sauyet S, Enright J, Neugebauer KM. 2022. The coilin N-terminus mediates multivalent interactions between coilin and Nopp140 to form and maintain Cajal bodies. Nat Commun 13: 6005. 10.1038/s41467-022-33434-2 36224177 PMC9556525

[GAD350518PUTC37] Darzacq X, Tjian R. 2022. Weak multivalent biomolecular interactions: a strength versus numbers tug of war with implications for phase partitioning. RNA 28: 48–51. 10.1261/rna.079004.121 34772790 PMC8675282

[GAD350518PUTC38] Das S, Singer RH, Yoon YJ. 2019. The travels of mRNAs in neurons: do they know where they are going? Curr Opin Neurobiol 57: 110–116. 10.1016/j.conb.2019.01.016 30784978 PMC6650148

[GAD350518PUTC39] Das S, Vera M, Gandin V, Singer RH, Tutucci E. 2021. Intracellular mRNA transport and localized translation. Nat Rev Mol Cell Biol 22: 483–504. 10.1038/s41580-021-00356-8 33837370 PMC9346928

[GAD350518PUTC40] Das S, Santos L, Failla AV, Ignatova Z. 2022. mRNAs sequestered in stress granules recover nearly completely for translation. RNA Biol 19: 877–884. 10.1080/15476286.2022.2094137 35796440 PMC9272840

[GAD350518PUTC41] Dasmeh P, Wagner A. 2021. Natural selection on the phase-separation properties of FUS during 160 MY of mammalian evolution. Mol Biol Evol 38: 940–951. 10.1093/molbev/msaa258 33022038 PMC7947763

[GAD350518PUTC42] Decker CJ, Teixeira D, Parker R. 2007. Edc3p and a glutamine/asparagine-rich domain of Lsm4p function in processing body assembly in *Saccharomyces cerevisiae*. J Cell Biol 179: 437–449. 10.1083/jcb.200704147 17984320 PMC2064791

[GAD350518PUTC43] Decker CJ, Burke JM, Mulvaney PK, Parker R. 2022. RNA is required for the integrity of multiple nuclear and cytoplasmic membrane-less RNP granules. EMBO J 41: e110137. 10.15252/embj.2021110137 35355287 PMC9058542

[GAD350518PUTC44] Delarue M, Brittingham GP, Pfeffer S, Surovtsev IV, Pinglay S, Kennedy KJ, Schaffer M, Gutierrez JI, Sang D, Poterewicz G, 2018. mTORC1 controls phase separation and the biophysical properties of the cytoplasm by tuning crowding. Cell 174: 338–349.e20. 10.1016/j.cell.2018.05.042 29937223 PMC10080728

[GAD350518PUTC45] Deryusheva S, Gall JG. 2009. Small Cajal body-specific RNAs of *Drosophila* function in the absence of cajal bodies. Mol Biol Cell 20: 5250–5259. 10.1091/mbc.e09-09-0777 19846657 PMC2793299

[GAD350518PUTC46] Dobrynin MA, Bashendjieva EO, Enukashvily NI. 2022. Germ granules in animal oogenesis. J Dev Biol 10: 43. 10.3390/jdb10040043 36278548 PMC9624338

[GAD350518PUTC47] Dodson AE, Kennedy S. 2020. Phase separation in germ cells and development. Dev Cell 55: 4–17. 10.1016/j.devcel.2020.09.004 33007213 PMC7572804

[GAD350518PUTC48] Eiermann N, Haneke K, Sun Z, Stoecklin G, Ruggieri A. 2020. Dance with the devil: stress granules and signaling in antiviral responses. Viruses 12: 984. 10.3390/v12090984 32899736 PMC7552005

[GAD350518PUTC49] Elaswad MT, Munderloh C, Watkins BM, Sharp KG, Breton E, Schisa JA. 2022a. Imaging-associated stress causes divergent phase transitions of RNA-binding proteins in the *Caenorhabditis elegans* germ line. G3 (Bethesda) 12: jkac172. 10.1093/g3journal/jkac172 35801939 PMC9434235

[GAD350518PUTC50] Elaswad MT, Watkins BM, Sharp KG, Munderloh C, Schisa JA. 2022b. Large RNP granules in *Caenorhabditis elegans* oocytes have distinct phases of RNA-binding proteins. G3 12: jkac173. 10.1093/g3journal/jkac173 35816006 PMC9434171

[GAD350518PUTC51] Elbaum-Garfinkle S, Kim Y, Szczepaniak K, Chen CC-H, Eckmann CR, Myong S, Brangwynne CP. 2015. The disordered P granule protein LAF-1 drives phase separation into droplets with tunable viscosity and dynamics. Proc Natl Acad Sci 112: 7189–7194. 10.1073/pnas.1504822112 26015579 PMC4466716

[GAD350518PUTC52] Elguindy MM, Mendell JT. 2021. NORAD-induced Pumilio phase separation is required for genome stability. Nature 595: 303–308. 10.1038/s41586-021-03633-w 34108682 PMC8266761

[GAD350518PUTC53] Elguindy MM, Kopp F, Goodarzi M, Rehfeld F, Thomas A, Chang T-C, Mendell JT. 2019. PUMILIO, but not RBMX, binding is required for regulation of genomic stability by noncoding RNA NORAD. Elife 8: e48625. 10.7554/eLife.48625 31343408 PMC6677556

[GAD350518PUTC54] Emenecker RJ, Holehouse AS, Strader LC. 2020. Emerging roles for phase separation in plants. Dev Cell 55: 69–83. 10.1016/j.devcel.2020.09.010 33049212 PMC7577370

[GAD350518PUTC55] Ephrussi A, Lehmann R. 1992. Induction of germ cell formation by oskar. Nature 358: 387–392. 10.1038/358387a0 1641021

[GAD350518PUTC56] Erdel F, Rippe K. 2018. Formation of chromatin subcompartments by phase separation. Biophys J 114: 2262–2270. 10.1016/j.bpj.2018.03.011 29628210 PMC6129460

[GAD350518PUTC57] Erdmann PS, Hou Z, Klumpe S, Khavnekar S, Beck F, Wilfling F, Plitzko JM, Baumeister W. 2021. In situ cryo-electron tomography reveals gradient organization of ribosome biogenesis in intact nucleoli. Nat Commun 12: 5364. 10.1038/s41467-021-25413-w 34508074 PMC8433212

[GAD350518PUTC58] Eulalio A, Behm-Ansmant I, Schweizer D, Izaurralde E. 2007. P-body formation is a consequence, not the cause, of RNA-mediated gene silencing. Mol Cell Biol 27: 3970–3981. 10.1128/MCB.00128-07 17403906 PMC1900022

[GAD350518PUTC59] Faber GP, Nadav-Eliyahu S, Shav-Tal Y. 2022. Nuclear speckles—a driving force in gene expression. J Cell Sci 135: jcs259594. 10.1242/jcs.259594 35788677 PMC9377712

[GAD350518PUTC60] Fare CM, Villani A, Drake LE, Shorter J. 2021. Higher-order organization of biomolecular condensates. Open Biol 11: 210137. 10.1098/rsob.210137 34129784 PMC8205532

[GAD350518PUTC61] Feric M, Vaidya N, Harmon TS, Mitrea DM, Zhu L, Richardson TM, Kriwacki RW, Pappu RV, Brangwynne CP. 2016. Coexisting liquid phases underlie nucleolar subcompartments. Cell 165: 1686–1697. 10.1016/j.cell.2016.04.047 27212236 PMC5127388

[GAD350518PUTC62] Ferrie JJ, Karr JP, Tjian R, Darzacq X. 2022. ‘Structure’–function relationships in eukaryotic transcription factors: the role of intrinsically disordered regions in gene regulation. Mol Cell 82: 3970–3984. 10.1016/j.molcel.2022.09.021 36265487

[GAD350518PUTC063] Fingerhut JM, Yamashita YM, 2020. mRNA localization mediates maturation of cytoplasmic cilia in *Drosophila* spermatogenesis. J Cell Biol 219: e202003084. 10.1083/jcb.202003084 32706373 PMC7480094

[GAD350518PUTC63] Folkmann AW, Putnam A, Lee CF, Seydoux G. 2021. Regulation of biomolecular condensates by interfacial protein clusters. Science 373: 1218–1224. 10.1126/science.abg7071 34516789 PMC8627561

[GAD350518PUTC64] Forman-Kay JD, Ditlev JA, Nosella ML, Lee HO. 2022. What are the distinguishing features and size requirements of biomolecular condensates and their implications for RNA-containing condensates? RNA 28: 36–47. 10.1261/rna.079026.121 34772786 PMC8675286

[GAD350518PUTC65] Franzmann TM, Jahnel M, Pozniakovsky A, Mahamid J, Holehouse AS, Nüske E, Richter D, Baumeister W, Grill SW, Pappu RV, 2018. Phase separation of a yeast prion protein promotes cellular fitness. Science 359: eaao5654. 10.1126/science.aao5654 29301985

[GAD350518PUTC66] Frottin F, Schueder F, Tiwary S, Gupta R, Körner R, Schlichthaerle T, Cox J, Jungmann R, Hartl FU, Hipp MS. 2019. The nucleolus functions as a phase-separated protein quality control compartment. Science 365: 342–347. 10.1126/science.aaw9157 31296649

[GAD350518PUTC67] Gallo CM, Munro E, Rasoloson D, Merritt C, Seydoux G. 2008. Processing bodies and germ granules are distinct RNA granules that interact in *C. elegans* embryos. Dev Biol 323: 76–87. 10.1016/j.ydbio.2008.07.008 18692039

[GAD350518PUTC68] Gao Z, Zhang W, Chang R, Zhang S, Yang G, Zhao G. 2021. Liquid–liquid phase separation: unraveling the enigma of biomolecular condensates in microbial cells. Front Microbiol 12: 751880. 10.3389/fmicb.2021.751880 34759902 PMC8573418

[GAD350518PUTC69] Girard C, Will CL, Peng J, Makarov EM, Kastner B, Lemm I, Urlaub H, Hartmuth K, Lührmann R. 2012. Post-transcriptional spliceosomes are retained in nuclear speckles until splicing completion. Nat Commun 3: 994. 10.1038/ncomms1998 22871813

[GAD350518PUTC70] Glauninger H, Hickernell CJW, Bard JAM, Drummond DA. 2022. Stressful steps: progress and challenges in understanding stress-induced mRNA condensation and accumulation in stress granules. Mol Cell 82: 2544–2556. 10.1016/j.molcel.2022.05.014 35662398 PMC9308734

[GAD350518PUTC71] Götte B, Panas MD, Hellström K, Liu L, Samreen B, Larsson O, Ahola T, McInerney GM. 2019. Separate domains of G3BP promote efficient clustering of alphavirus replication complexes and recruitment of the translation initiation machinery. PLoS Pathog 15: e1007842. 10.1371/journal.ppat.1007842 31199850 PMC6594655

[GAD350518PUTC72] Gouveia B, Kim Y, Shaevitz JW, Petry S, Stone HA, Brangwynne CP. 2022. Capillary forces generated by biomolecular condensates. Nature 609: 255–264. 10.1038/s41586-022-05138-6 36071192

[GAD350518PUTC73] Greenblatt EJ, Spradling AC. 2018. Fragile X mental retardation 1 gene enhances the translation of large autism-related proteins. Science 361: 709–712. 10.1126/science.aas9963 30115809 PMC6905618

[GAD350518PUTC74] Guillén-Boixet J, Kopach A, Holehouse AS, Wittmann S, Jahnel M, Schlüßler R, Kim K, Trussina IREA, Wang J, Mateju D, 2020. RNA-induced conformational switching and clustering of G3BP drive stress granule assembly by condensation. Cell 181: 346–361.e17. 10.1016/j.cell.2020.03.049 32302572 PMC7181197

[GAD350518PUTC75] Guo C, Luo Z, Lin C. 2022. Phase separation properties in transcriptional organization. Biochemistry 61: 2456–2460. 10.1021/acs.biochem.2c00220 35950649

[GAD350518PUTC76] Hampoelz B, Schwarz A, Ronchi P, Bragulat-Teixidor H, Tischer C, Gaspar I, Ephrussi A, Schwab Y, Beck M. 2019. Nuclear pores assemble from nucleoporin condensates during oogenesis. Cell 179: 671–686.e17. 10.1016/j.cell.2019.09.022 31626769 PMC6838685

[GAD350518PUTC77] Hanazawa M, Yonetani M, Sugimoto A. 2011. PGL proteins self associate and bind RNPs to mediate germ granule assembly in *C. elegans*. J Cell Biol 192: 929–937. 10.1083/jcb.201010106 21402787 PMC3063142

[GAD350518PUTC78] He X, Yuan J, Wang Y. 2021. G3BP1 binds to guanine quadruplexes in mRNAs to modulate their stabilities. Nucleic Acids Res 49: 11323–11336. 10.1093/nar/gkab873 34614161 PMC8565330

[GAD350518PUTC79] Hernandez-Verdun D, Roussel P, Thiry M, Sirri V, Lafontaine DLJ. 2010. The nucleolus: structure/function relationship in RNA metabolism. WIREs RNA 1: 415–431. 10.1002/wrna.39 21956940

[GAD350518PUTC80] Hilliker A, Gao Z, Jankowsky E, Parker R. 2011. The DEAD-box protein Ded1 modulates translation by the formation and resolution of an eIF4F–mRNA complex. Mol Cell 43: 962–972. 10.1016/j.molcel.2011.08.008 21925384 PMC3268518

[GAD350518PUTC81] Hirose T, Virnicchi G, Tanigawa A, Naganuma T, Li R, Kimura H, Yokoi T, Nakagawa S, Bénard M, Fox AH, 2014. NEAT1 long noncoding RNA regulates transcription via protein sequestration within subnuclear bodies. Mol Biol Cell 25: 169–183. 10.1091/mbc.e13-09-0558 24173718 PMC3873887

[GAD350518PUTC82] Hirose T, Ninomiya K, Nakagawa S, Yamazaki T. 2023. A guide to membraneless organelles and their various roles in gene regulation. Nat Rev Mol Cell Biol 24: 288–304. 10.1038/s41580-022-00558-8 36424481

[GAD350518PUTC83] Hnisz D, Shrinivas K, Young RA, Chakraborty AK, Sharp PA. 2017. A phase separation model for transcriptional control. Cell 169: 13–23. 10.1016/j.cell.2017.02.007 28340338 PMC5432200

[GAD350518PUTC84] Hochberg-Laufer H, Neufeld N, Brody Y, Nadav-Eliyahu S, Ben-Yishay R, Shav-Tal Y. 2019. Availability of splicing factors in the nucleoplasm can regulate the release of mRNA from the gene after transcription. PLoS Genet 15: e1008459. 10.1371/journal.pgen.1008459 31765392 PMC6901260

[GAD350518PUTC85] Hondele M, Sachdev R, Heinrich S, Wang J, Vallotton P, Fontoura BMA, Weis K. 2019. DEAD-box ATPases are global regulators of phase-separated organelles. Nature 573: 144–148. 10.1038/s41586-019-1502-y 31435012 PMC7617057

[GAD350518PUTC86] Hong S, Choi S, Kim R, Koh J. 2020. Mechanisms of macromolecular interactions mediated by protein intrinsic disorder. Mol Cells 43: 899–908. 10.14348/molcells.2020.0186 33243935 PMC7700844

[GAD350518PUTC87] Hori Y, Engel C, Kobayashi T. 2023. Regulation of ribosomal RNA gene copy number, transcription and nucleolus organization in eukaryotes. Nat Rev Mol Cell Biol 10.1038/s41580-022-00573-9 36732602

[GAD350518PUTC88] Horvathova I, Voigt F, Kotrys AV, Zhan Y, Artus-Revel CG, Eglinger J, Stadler MB, Giorgetti L, Chao JA. 2017. The dynamics of mRNA turnover revealed by single-molecule imaging in single cells. Mol Cell 68: 615–625.e9. 10.1016/j.molcel.2017.09.030 29056324

[GAD350518PUTC89] Hubstenberger A, Courel M, Bénard M, Souquere S, Ernoult-Lange M, Chouaib R, Yi Z, Morlot J-B, Munier A, Fradet M, 2017. P-body purification reveals the condensation of repressed mRNA regulons. Mol Cell 68: 144–157.e5. 10.1016/j.molcel.2017.09.003 28965817

[GAD350518PUTC90] Hyman AA, Weber CA, Jülicher F. 2014. Liquid–liquid phase separation in biology. Annu Rev Cell Dev Biol 30: 39–58. 10.1146/annurev-cellbio-100913-013325 25288112

[GAD350518PUTC91] Irgen-Gioro S, Yoshida S, Walling V, Chong S. 2022. Fixation can change the appearance of phase separation in living cells. Elife 11: e79903. 10.7554/eLife.79903 36444977 PMC9817179

[GAD350518PUTC92] Iserman C, Desroches Altamirano C, Jegers C, Friedrich U, Zarin T, Fritsch AW, Mittasch M, Domingues A, Hersemann L, Jahnel M, 2020. Condensation of Ded1p promotes a translational switch from housekeeping to stress protein production. Cell 181: 818–831.e19. 10.1016/j.cell.2020.04.009 32359423 PMC7237889

[GAD350518PUTC93] Israelachvili JN. 2011. Thermodynamic principles of self-assembly. In Intermolecular and surface forces, 3^rd^ ed (ed. Israelachvili JN), pp. 503–534. Academic Press, San Diego

[GAD350518PUTC94] Jawerth L, Fischer-Friedrich E, Saha S, Wang J, Franzmann T, Zhang X, Sachweh J, Ruer M, Ijavi M, Saha S, 2020. Protein condensates as aging Maxwell fluids. Science 370: 1317–1323. 10.1126/science.aaw4951 33303613

[GAD350518PUTC95] Jiang L, Shao C, Wu Q-J, Chen G, Zhou J, Yang B, Li H, Gou L-T, Zhang Y, Wang Y, 2017. NEAT1 scaffolds RNA-binding proteins and the microprocessor to globally enhance pri-miRNA processing. Nat Struct Mol Biol 24: 816–824. 10.1038/nsmb.3455 28846091 PMC5766049

[GAD350518PUTC096] Jiang Y, Pyo A, Brangwynne CP, Stone HA, Wingreen NS, Zhang Y. 2023. Interface resistance of biomolecular condensates. Biophys J 122: 442a. 10.1016/j.bpj.2022.11.238836403088

[GAD350518PUTC96] Johnson C, Primorac D, McKinstry M, McNeil J, Rowe D, Lawrence JB. 2000. Tracking Col1a1 RNA in osteogenesis imperfecta: splice-defective transcripts initiate transport from the gene but are retained within the Sc35 domain. J Cell Biol 150: 417–432. 10.1083/jcb.150.3.417 10931857 PMC2175183

[GAD350518PUTC97] Jud M, Razelun J, Bickel J, Czerwinski M, Schisa JA. 2007. Conservation of large foci formation in arrested oocytes of *Caenorhabditis* nematodes. Dev Genes Evol 217: 221–226. 10.1007/s00427-006-0130-3 17216268

[GAD350518PUTC98] Kar M, Dar F, Welsh TJ, Vogel LT, Kühnemuth R, Majumdar A, Krainer G, Franzmann TM, Alberti S, Seidel CAM, 2022. Phase-separating RNA-binding proteins form heterogeneous distributions of clusters in subsaturated solutions. Proc Natl Acad Sci 119: e2202222119. 10.1073/pnas.2202222119 35787038 PMC9282234

[GAD350518PUTC99] Kato M, Yang Y-S, Sutter BM, Wang Y, McKnight SL, Tu BP. 2019. Redox state controls phase separation of the yeast ataxin-2 protein via reversible oxidation of its methionine-rich low-complexity domain. Cell 177: 711–721.e8. 10.1016/j.cell.2019.02.044 30982603 PMC6752730

[GAD350518PUTC100] Kato M, Zhou X, McKnight SL. 2022. How do protein domains of low sequence complexity work? RNA 28: 3–15. 10.1261/rna.078990.121 34670847 PMC8675291

[GAD350518PUTC101] Kedersha N, Anderson P. 2002. Stress granules: sites of mRNA triage that regulate mRNA stability and translatability. Biochem Soc Trans 30: 963–969. 10.1042/bst0300963 12440955

[GAD350518PUTC102] Kedersha N, Stoecklin G, Ayodele M, Yacono P, Lykke-Andersen J, Fritzler MJ, Scheuner D, Kaufman RJ, Golan DE, Anderson P. 2005. Stress granules and processing bodies are dynamically linked sites of mRNP remodeling. J Cell Biol 169: 871–884. 10.1083/jcb.200502088 15967811 PMC2171635

[GAD350518PUTC103] Kedersha N, Panas MD, Achorn CA, Lyons S, Tisdale S, Hickman T, Thomas M, Lieberman J, McInerney GM, Ivanov P, 2016. G3BP–Caprin1–USP10 complexes mediate stress granule condensation and associate with 40S subunits. J Cell Biol 212: 845. 10.1083/jcb.201508028 27022092 PMC4810302

[GAD350518PUTC104] Khong A, Parker R. 2018. mRNP architecture in translating and stress conditions reveals an ordered pathway of mRNP compaction. J Cell Biol 217: 4124–4140. 10.1083/jcb.201806183 30322972 PMC6279387

[GAD350518PUTC105] Khong A, Matheny T, Jain S, Mitchell SF, Wheeler JR, Parker R. 2017. The stress granule transcriptome reveals principles of mRNA accumulation in stress granules. Mol Cell 68: 808–820.e5. 10.1016/j.molcel.2017.10.015 29129640 PMC5728175

[GAD350518PUTC106] Kim J, Venkata NC, Hernandez Gonzalez GA, Khanna N, Belmont AS. 2019a. Gene expression amplification by nuclear speckle association. J Cell Biol 219: e201904046. 10.1083/jcb.201904046 PMC7039209 31757787

[GAD350518PUTC107] Kim TH, Tsang B, Vernon RM, Sonenberg N, Kay LE, Forman-Kay JD. 2019b. Phospho-dependent phase separation of FMRP and CAPRIN1 recapitulates regulation of translation and deadenylation. Science 365: 825–829. 10.1126/science.aax4240 31439799

[GAD350518PUTC108] Lacroix E, Audas TE. 2022. Keeping up with the condensates: the retention, gain, and loss of nuclear membrane-less organelles. Front Mol Biosci 9: 998363. 10.3389/fmolb.2022.998363 36203874 PMC9530788

[GAD350518PUTC109] Lafontaine DLJ, Riback JA, Bascetin R, Brangwynne CP. 2021. The nucleolus as a multiphase liquid condensate. Nat Rev Mol Cell Biol 22: 165–182. 10.1038/s41580-020-0272-6 32873929

[GAD350518PUTC110] Langdon EM, Qiu Y, Niaki AG, McLaughlin GA, Weidmann CA, Gerbich TM, Smith JA, Crutchley JM, Termini CM, Weeks KM, 2018. mRNA structure determines specificity of a polyQ-driven phase separation. Science 360: 922–927. 10.1126/science.aar7432 29650703 PMC6192030

[GAD350518PUTC111] Laver JD, Ly J, Winn AK, Karaiskakis A, Lin S, Nie K, Benic G, Jaberi-Lashkari N, Cao WX, Khademi A, 2020. The RNA-binding protein rasputin/G3BP enhances the stability and translation of its target mRNAs. Cell Rep 30: 3353–3367.e7. 10.1016/j.celrep.2020.02.066 32160542

[GAD350518PUTC112] Lawrimore J, Kolbin D, Stanton J, Khan M, de Larminat SC, Lawrimore C, Yeh E, Bloom K. 2021. The rDNA is biomolecular condensate formed by polymer–polymer phase separation and is sequestered in the nucleolus by transcription and R-loops. Nucleic Acids Res 49: 4586–4598. 10.1093/nar/gkab229 33836082 PMC8096216

[GAD350518PUTC113] Lee C, Occhipinti P, Gladfelter AS. 2015. PolyQ-dependent RNA–protein assemblies control symmetry breaking. J Cell Biol 208: 533–544. 10.1083/jcb.201407105 25713414 PMC4347642

[GAD350518PUTC114] Lee CYS, Putnam A, Lu T, He SX, Ouyang JPT, Seydoux G. 2020. Recruitment of mRNAs to P granules by condensation with intrinsically-disordered proteins. Elife 9: e52896. 10.7554/eLife.52896 31975687 PMC7007223

[GAD350518PUTC115] Leidescher S, Ribisel J, Ullrich S, Feodorova Y, Hildebrand E, Galitsyna A, Bultmann S, Link S, Thanisch K, Mulholland C, 2022. Spatial organization of transcribed eukaryotic genes. Nat Cell Biol 24: 327–339. 10.1038/s41556-022-00847-6 35177821 PMC9380065

[GAD350518PUTC116] Li P, Banjade S, Cheng H-C, Kim S, Chen B, Guo L, Llaguno M, Hollingsworth JV, King DS, Banani SF, 2012. Phase transitions in the assembly of multivalent signalling proteins. Nature 483: 336–340. 10.1038/nature10879 22398450 PMC3343696

[GAD350518PUTC117] Liao Y-C, Fernandopulle MS, Wang G, Choi H, Hao L, Drerup CM, Patel R, Qamar S, Nixon-Abell J, Shen Y, 2019. RNA granules hitchhike on lysosomes for long-distance transport, using annexin a11 as a molecular tether. Cell 179: 147–164.e20. 10.1016/j.cell.2019.08.050 31539493 PMC6890474

[GAD350518PUTC118] Lin S, Rajan S, Lemberg S, Altawil M, Anderson K, Bryant R, Cappeta S, Chin B, Hamdan I, Hamer A, 2022. Production of nascent ribosome precursors within the nucleolar microenvironment of *Saccharomyces cerevisiae*. Genetics 221: iyac070. 10.1093/genetics/iyac070 35657327 PMC9252279

[GAD350518PUTC119] Ling SHM, Decker CJ, Walsh MA, She M, Parker R, Song H. 2008. Crystal structure of human Edc3 and its functional implications. Mol Cell Biol 28: 5965–5976. 10.1128/MCB.00761-08 18678652 PMC2547010

[GAD350518PUTC120] Lyon AS, Peeples WB, Rosen MK. 2021. A framework for understanding the functions of biomolecular condensates across scales. Nat Rev Mol Cell Biol 22: 215–235. 10.1038/s41580-020-00303-z 33169001 PMC8574987

[GAD350518PUTC121] Lyons H, Veettil RT, Pradhan P, Fornero C, De La Cruz N, Ito K, Eppert M, Roeder RG, Sabari BR. 2023. Functional partitioning of transcriptional regulators by patterned charge blocks. Cell 186: 327–345.e28. 10.1016/j.cell.2022.12.013 36603581 PMC9910284

[GAD350518PUTC122] Ma W, Mayr C. 2018. A membraneless organelle associated with the endoplasmic reticulum enables 3′UTR-mediated protein–protein interactions. Cell 175: 1492–1506.e19. 10.1016/j.cell.2018.10.007 30449617 PMC6711188

[GAD350518PUTC123] Ma W, Zhen G, Xie W, Mayr C. 2021. In vivo reconstitution finds multivalent RNA–RNA interactions as drivers of mesh-like condensates. Elife 10: e64252. 10.7554/eLife.64252 33650968 PMC7968931

[GAD350518PUTC124] Maharana S, Wang J, Papadopoulos DK, Richter D, Pozniakovsky A, Poser I, Bickle M, Rizk S, Guillén-Boixet J, Franzmann TM, 2018. RNA buffers the phase separation behavior of prion-like RNA binding proteins. Science 360: 918–921. 10.1126/science.aar7366 29650702 PMC6091854

[GAD350518PUTC125] Mahowald AP. 1968. Polar granules of *Drosophila*. II. Ultrastructural changes during early embryogenesis. J Exp Zool 167: 237–261. 10.1002/jez.1401670211 5676182

[GAD350518PUTC126] Manage KI, Rogers AK, Wallis DC, Uebel CJ, Anderson DC, Nguyen DAH, Arca K, Brown KC, Cordeiro Rodrigues RJ, de Albuquerque BFM, 2020. A tudor domain protein, SIMR-1, promotes siRNA production at piRNA-targeted mRNAs in *C. elegans*. Elife 9: e56731. 10.7554/eLife.56731 32338603 PMC7255803

[GAD350518PUTC127] Mangan H, McStay B. 2021. Human nucleoli comprise multiple constrained territories, tethered to individual chromosomes. Genes Dev 35: 483–488. 10.1101/gad.348234.121 33664058 PMC8015717

[GAD350518PUTC128] Markmiller S, Soltanieh S, Server KL, Mak R, Jin W, Fang MY, Luo E-C, Krach F, Yang D, Sen A, 2018. Context-dependent and disease-specific diversity in protein interactions within stress granules. Cell 172: 590–604.e13. 10.1016/j.cell.2017.12.032 29373831 PMC5969999

[GAD350518PUTC129] Marnik EA, Fuqua JH, Sharp CS, Rochester JD, Xu EL, Holbrook SE, Updike DL. 2019. Germline maintenance through the multifaceted activities of GLH/Vasa in *Caenorhabditis elegans* P granules. Genetics 213: 923–939. 10.1534/genetics.119.302670 31506335 PMC6827368

[GAD350518PUTC130] Martin EW, Holehouse AS. 2020. Intrinsically disordered protein regions and phase separation: sequence determinants of assembly or lack thereof. Emerg Top Life Sci 4: 307–329. 10.1042/ETLS20190164 33078839

[GAD350518PUTC131] Mateju D, Chao JA. 2022. Stress granules: regulators or by-products? FEBS J 289: 363–373. 10.1111/febs.15821 33725420

[GAD350518PUTC132] Mateju D, Eichenberger B, Voigt F, Eglinger J, Roth G, Chao JA. 2020. Single-molecule imaging reveals translation of mRNAs localized to stress granules. Cell 183: 1801–1812.e13. 10.1016/j.cell.2020.11.010 33308477

[GAD350518PUTC133] Mazroui R, Huot M-E, Tremblay S, Filion C, Labelle Y, Khandjian EW. 2002. Trapping of messenger RNA by fragile X mental retardation protein into cytoplasmic granules induces translation repression. Hum Mol Genet 11: 3007–3017. 10.1093/hmg/11.24.3007 12417522

[GAD350518PUTC134] McSwiggen DT, Hansen AS, Teves SS, Marie-Nelly H, Hao Y, Heckert AB, Umemoto KK, Dugast-Darzacq C, Tjian R, Darzacq X. 2019a. Evidence for DNA-mediated nuclear compartmentalization distinct from phase separation. Elife 8: e47098. 10.7554/eLife.47098 31038454 PMC6522219

[GAD350518PUTC135] McSwiggen DT, Mir M, Darzacq X, Tjian R. 2019b. Evaluating phase separation in live cells: diagnosis, caveats, and functional consequences. Genes Dev 33: 1619–1634. 10.1101/gad.331520.119 31594803 PMC6942051

[GAD350518PUTC136] Misteli T, Cáceres JF, Spector DL. 1997. The dynamics of a pre-mRNA splicing factor in living cells. Nature 387: 523–527. 10.1038/387523a0 9168118

[GAD350518PUTC137] Mitrea DM, Cika JA, Guy CS, Ban D, Banerjee PR, Stanley CB, Nourse A, Deniz AA, Kriwacki RW. 2016. Nucleophosmin integrates within the nucleolus via multi-modal interactions with proteins displaying R-rich linear motifs and rRNA. Elife 5: e13571. 10.7554/eLife.13571 26836305 PMC4786410

[GAD350518PUTC138] Mittag T, Pappu RV. 2022. A conceptual framework for understanding phase separation and addressing open questions and challenges. Mol Cell 82: 2201–2214. 10.1016/j.molcel.2022.05.018 35675815 PMC9233049

[GAD350518PUTC139] Mollet S, Cougot N, Wilczynska A, Dautry F, Kress M, Bertrand E, Weil D. 2008. Translationally repressed mRNA transiently cycles through stress granules during stress. Mol Biol Cell 19: 4469–4479. 10.1091/mbc.E08-05-0499 18632980 PMC2555929

[GAD350518PUTC140] Moon SL, Morisaki T, Khong A, Lyon K, Parker R, Stasevich TJ. 2019. Multicolor single-molecule tracking of mRNA interactions with RNP granules. Nat Cell Biol 21: 162–168. 10.1038/s41556-018-0263-4 30664789 PMC6375083

[GAD350518PUTC141] Morales-Polanco F, Bates C, Lui J, Casson J, Solari CA, Pizzinga M, Forte G, Griffin C, Garner KEL, Burt HE, 2021. Core fermentation (CoFe) granules focus coordinated glycolytic mRNA localization and translation to fuel glucose fermentation. iScience 24: 102069. 10.1016/j.isci.2021.102069 33554071 PMC7859310

[GAD350518PUTC142] Mugler CF, Hondele M, Heinrich S, Sachdev R, Vallotton P, Koek AY, Chan LY, Weis K. 2016. ATPase activity of the DEAD-box protein Dhh1 controls processing body formation. Elife 5: e18746. 10.7554/eLife.18746 27692063 PMC5096884

[GAD350518PUTC143] Munschauer M, Nguyen CT, Sirokman K, Hartigan CR, Hogstrom L, Engreitz JM, Ulirsch JC, Fulco CP, Subramanian V, Chen J, 2018. The NORAD lncRNA assembles a topoisomerase complex critical for genome stability. Nature 561: 132–136. 10.1038/s41586-018-0453-z 30150775

[GAD350518PUTC144] Murthy AC, Dignon GL, Kan Y, Zerze GH, Parekh SH, Mittal J, Fawzi NL. 2019. Molecular interactions underlying liquid–liquid phase separation of the FUS low-complexity domain. Nat Struct Mol Biol 26: 637–648. 10.1038/s41594-019-0250-x 31270472 PMC6613800

[GAD350518PUTC145] Musacchio A. 2022. On the role of phase separation in the biogenesis of membraneless compartments. EMBO J 41: e109952. 10.15252/embj.2021109952 35107832 PMC8886532

[GAD350518PUTC146] Nakagawa S, Shimada M, Yanaka K, Mito M, Arai T, Takahashi E, Fujita Y, Fujimori T, Standaert L, Marine J-C, 2014. The lncRNA Neat1 is required for corpus luteum formation and the establishment of pregnancy in a subpopulation of mice. Development 141: 4618–4627. 10.1242/dev.110544 25359727 PMC4302932

[GAD350518PUTC147] Nakagawa K, Narayanan K, Wada M, Makino S. 2018. Inhibition of stress granule formation by middle east respiratory syndrome coronavirus 4a accessory protein facilitates viral translation, leading to efficient virus replication. J Virol 92: e00902-18. 10.1128/JVI.00902-18 30068649 PMC6158436

[GAD350518PUTC148] Nakano SI, Kirihata T, Fujii S, Sakai H, Kuwahara M, Sawai H, Sugimoto N. 2007. Influence of cationic molecules on the hairpin to duplex equilibria of self-complementary DNA and RNA oligonucleotides. Nucleic Acids Res 35: 486–494. 10.1093/nar/gkl1073 17169988 PMC1802612

[GAD350518PUTC149] Nedelsky NB, Taylor JP. 2022. Pathological phase transitions in ALS-FTD impair dynamic RNA–protein granules. RNA 28: 97–113. 10.1261/rna.079001.121 34706979 PMC8675280

[GAD350518PUTC150] Németh A, Grummt I. 2018. Dynamic regulation of nucleolar architecture. Curr Opin Cell Biol 52: 105–111. 10.1016/j.ceb.2018.02.013 29529563

[GAD350518PUTC151] Niepielko MG, Eagle WVI, Gavis ER. 2018. Stochastic seeding coupled with mRNA self-recruitment generates heterogeneous *Drosophila* germ granules. Curr Biol 28: 1872–1881.e3. 10.1016/j.cub.2018.04.037 29861136 PMC6008217

[GAD350518PUTC152] Nott TJ, Petsalaki E, Farber P, Jervis D, Fussner E, Plochowietz A, Craggs TD, Bazett-Jones DP, Pawson T, Forman-Kay JD, 2015. Phase transition of a disordered nuage protein generates environmentally responsive membraneless organelles. Mol Cell 57: 936–947. 10.1016/j.molcel.2015.01.013 25747659 PMC4352761

[GAD350518PUTC153] Nott TJ, Craggs TD, Baldwin AJ. 2016. Membraneless organelles can melt nucleic acid duplexes and act as biomolecular filters. Nat Chem 8: 569–575. 10.1038/nchem.2519 27219701

[GAD350518PUTC154] Ohn T, Kedersha N, Hickman T, Tisdale S, Anderson P. 2008. A functional RNAi screen links O-GlcNAc modification of ribosomal proteins to stress granule and processing body assembly. Nat Cell Biol 10: 1224–1231. 10.1038/ncb1783 18794846 PMC4318256

[GAD350518PUTC155] Onuchic PL, Milin AN, Alshareedah I, Deniz AA, Banerjee PR. 2019. Divalent cations can control a switch-like behavior in heterotypic and homotypic RNA coacervates. Sci Rep 9: 12161. 10.1038/s41598-019-48457-x 31434954 PMC6704260

[GAD350518PUTC156] Ouyang JPT, Seydoux G. 2022. Nuage condensates: accelerators or circuit breakers for sRNA silencing pathways? RNA 28: 58–66. 10.1261/rna.079003.121 34772788 PMC8675287

[GAD350518PUTC157] Ouyang JPT, Zhang WL, Seydoux G. 2022. The conserved helicase ZNFX-1 memorializes silenced RNAs in perinuclear condensates. Nat Cell Biol 24: 1129–1140. 10.1038/s41556-022-00940-w 35739318 PMC9276528

[GAD350518PUTC158] Palacio M, Taatjes DJ. 2022. Merging established mechanisms with new insights: condensates, hubs, and the regulation of RNA polymerase II transcription. J Mol Biol 434: 167216. 10.1016/j.jmb.2021.167216 34474085 PMC8748285

[GAD350518PUTC159] Parker DM, Winkenbach LP, Boyson S, Saxton MN, Daidone C, Al-Mazaydeh ZA, Nishimura MT, Mueller F, Nishimura EO. 2020. mRNA localization is linked to translation regulation in the *Caenorhabditis elegans* germ lineage. Development 147: dev186817. 10.1242/dev.186817 32541012 PMC7358130

[GAD350518PUTC160] Parker DM, Winkenbach LP, Osborne Nishimura E. 2022. It's just a phase: exploring the relationship between mRNA, biomolecular condensates, and translational control. Front Genet 13. 10.3389/fgene.2022.931220 PMC9271857 35832192

[GAD350518PUTC161] Parry BR, Surovtsev IV, Cabeen MT, O'Hern CS, Dufresne ER, Jacobs-Wagner C. 2014. The bacterial cytoplasm has glass-like properties and is fluidized by metabolic activity. Cell 156: 183–194. 10.1016/j.cell.2013.11.028 24361104 PMC3956598

[GAD350518PUTC162] Patange S, Ball DA, Karpova TS, Larson DR. 2021. Towards a ‘spot on’ understanding of transcription in the nucleus. J Mol Biol 433: 167016. 10.1016/j.jmb.2021.167016 33951451 PMC8184600

[GAD350518PUTC163] Patterson JR, Wood MP, Schisa JA. 2011. Assembly of RNP granules in stressed and aging oocytes requires nucleoporins and is coordinated with nuclear membrane blebbing. Dev Biol 353: 173–185. 10.1016/j.ydbio.2011.02.028 21382369 PMC3096477

[GAD350518PUTC164] Peran I, Mittag T. 2020. Molecular structure in biomolecular condensates. Curr Opin Struct Biol 60: 17–26. 10.1016/j.sbi.2019.09.007 31790873 PMC7117980

[GAD350518PUTC165] Pham K, Masoudi N, Leyva-Díaz E, Hobert O. 2021. A nervous system-specific subnuclear organelle in *Caenorhabditis elegans*. Genetics 217: 1–17. 10.1093/genetics/iyaa016 PMC8045701 33683371

[GAD350518PUTC166] Phillips CM, Updike DL. 2022. Germ granules and gene regulation in the *Caenorhabditis elegans* germline. Genetics 220: iyab195. 10.1093/genetics/iyab195 35239965 PMC8893257

[GAD350518PUTC167] Phillips CM, Montgomery TA, Breen PC, Ruvkun G. 2012. MUT-16 promotes formation of perinuclear Mutator foci required for RNA silencing in the *C. elegans* germline. Genes Dev 26: 1433–1444. 10.1101/gad.193904.112 22713602 PMC3403012

[GAD350518PUTC168] Pizzinga M, Bates C, Lui J, Forte G, Morales-Polanco F, Linney E, Knotkova B, Wilson B, Solari CA, Berchowitz LE, 2019. Translation factor mRNA granules direct protein synthetic capacity to regions of polarized growth. J Cell Biol 218: 1564–1581. 10.1083/jcb.201704019 30877141 PMC6504908

[GAD350518PUTC169] Pontius BW. 1993. Close encounters: why unstructured, polymeric domains can increase rates of specific macromolecular association. Trends Biochem Sci 18: 181–186. 10.1016/0968-0004(93)90111-Y 8328018

[GAD350518PUTC170] Poudyal RR, Guth-Metzler RM, Veenis AJ, Frankel EA, Keating CD, Bevilacqua PC. 2019. Template-directed RNA polymerization and enhanced ribozyme catalysis inside membraneless compartments formed by coacervates. Nat Commun 10: 490. 10.1038/s41467-019-08353-4 30700721 PMC6353945

[GAD350518PUTC171] Pritišanac I, Zarin T, Forman-Kay JD, Moses AM. 2020. Whence blobs? phylogenetics of functional protein condensates. Biochem Soc Trans 48: 2151–2158. 10.1042/BST20200355 32985656

[GAD350518PUTC172] Putnam AA, Jankowsky E. 2013. DEAD-box helicases as integrators of RNA, nucleotide and protein binding. Biochim Biophys Acta 1829: 884–893. 10.1016/j.bbagrm.2013.02.002 23416748 PMC3661757

[GAD350518PUTC173] Putnam A, Seydoux G. 2023. Intrinsically disordered regions: a platform for regulated assembly of biomolecular condensates. In Droplets of life (ed. Uversky VN), pp. 397–430. Academic Press, Amsterdam, the Netherlands.

[GAD350518PUTC174] Putnam A, Cassani M, Smith J, Seydoux G. 2019. A gel phase promotes condensation of liquid P granules in *Caenorhabditis elegans* embryos. Nat Struct Mol Biol 26: 220–226. 10.1038/s41594-019-0193-2 30833787 PMC6668929

[GAD350518PUTC175] Quinodoz SA, Ollikainen N, Tabak B, Palla A, Schmidt JM, Detmar E, Lai MM, Shishkin AA, Bhat P, Takei Y, 2018. Higher-order inter-chromosomal hubs shape 3D genome organization in the nucleus. Cell 174: 744–757.e24. 10.1016/j.cell.2018.05.024 29887377 PMC6548320

[GAD350518PUTC176] Rangan P, DeGennaro M, Jaime-Bustamante K, Coux R-X, Martinho RG, Lehmann R. 2009. Temporal and spatial control of germ-plasm RNAs. Curr Biol 19: 72–77. 10.1016/j.cub.2008.11.066 19110432 PMC2766415

[GAD350518PUTC177] Ranganathan S, Shakhnovich EI. 2020. Dynamic metastable long-living droplets formed by sticker-spacer proteins. Elife 9: e56159. 10.7554/eLife.56159 32484438 PMC7360371

[GAD350518PUTC178] Rao BS, Parker R. 2017. Numerous interactions act redundantly to assemble a tunable size of P bodies in *Saccharomyces cerevisiae*. Proc Natl Acad Sci 114: E9569–E9578. 10.1073/pnas.1712396114 29078371 PMC5692575

[GAD350518PUTC179] Reineke LC, Lloyd RE. 2015. The stress granule protein G3BP1 recruits protein kinase R to promote multiple innate immune antiviral responses. J Virol 89: 2575–2589. 10.1128/JVI.02791-14 25520508 PMC4325707

[GAD350518PUTC180] Reineke LC, Kedersha N, Langereis MA, van Kuppeveld FJM, Lloyd RE. 2015. Stress granules regulate double-stranded RNA-dependent protein kinase activation through a complex containing G3BP1 and Caprin1. MBio 6: e02486-14. 10.1128/mBio.02486-14 25784705 PMC4453520

[GAD350518PUTC181] Rhine K, Vidaurre V, Myong S. 2020. RNA droplets. Annu Rev Biophys 49: 247–265. 10.1146/annurev-biophys-052118-115508 32040349 PMC7695521

[GAD350518PUTC182] Riback JA, Katanski CD, Kear-Scott JL, Pilipenko EV, Rojek AE, Sosnick TR, Drummond DA. 2017. Stress-triggered phase separation is an adaptive, evolutionarily tuned response. Cell 168: 1028–1040.e19. 10.1016/j.cell.2017.02.027 28283059 PMC5401687

[GAD350518PUTC183] Riback JA, Zhu L, Ferrolino MC, Tolbert M, Mitrea DM, Sanders DW, Wei M-T, Kriwacki RW, Brangwynne CP. 2020. Composition-dependent thermodynamics of intracellular phase separation. Nature 581: 209–214. 10.1038/s41586-020-2256-2 32405004 PMC7733533

[GAD350518PUTC184] Riback JA, Eeftens JM, Lee DSW, Quinodoz SA, Beckers L, Becker LA, Brangwynne CP. 2022. Viscoelastic RNA entanglement and advective flow underlie nucleolar form and function. bioRxiv 10.1101/2021.12.31.474660

[GAD350518PUTC185] Ripin N, Parker R. 2022. Are stress granules the RNA analogs of misfolded protein aggregates? RNA 28: 67–75. 10.1261/rna.079000.121 34670846 PMC8675284

[GAD350518PUTC186] Rippe K, Papantonis A. 2022. Functional organization of RNA polymerase II in nuclear subcompartments. Curr Opin Cell Biol 74: 88–96. 10.1016/j.ceb.2022.01.005 35217398

[GAD350518PUTC187] Roden C, Gladfelter AS. 2021. RNA contributions to the form and function of biomolecular condensates. Nat Rev Mol Cell Biol 22: 183–195. 10.1038/s41580-020-0264-6 32632317 PMC7785677

[GAD350518PUTC188] Rodríguez-Nuevo A, Torres-Sanchez A, Duran JM, De Guirior C, Martínez-Zamora MA, Böke E. 2022. Oocytes maintain ROS-free mitochondrial metabolism by suppressing complex I. Nature 607: 756–761. 10.1038/s41586-022-04979-5 35859172 PMC9329100

[GAD350518PUTC189] Rostam N, Ghosh S, Chow CFW, Hadarovich A, Landerer C, Ghosh R, Moon HK, Hersemann L, Mitrea DM, Klein IA, 2023. CD-CODE: crowdsourcing condensate database and encyclopedia. Nat Methods 10.1038/s41592-023-01831-0 PMC10172118 37024650

[GAD350518PUTC190] Saha S, Weber CA, Nousch M, Adame-Arana O, Hoege C, Hein MY, Osborne-Nishimura E, Mahamid J, Jahnel M, Jawerth L, 2016. Polar positioning of phase-separated liquid compartments in cells regulated by an mRNA competition mechanism. Cell 166: 1572–1584.e16. 10.1016/j.cell.2016.08.006 27594427 PMC5034880

[GAD350518PUTC191] Sanders DW, Kedersha N, Lee DSW, Strom AR, Drake V, Riback JA, Bracha D, Eeftens JM, Iwanicki A, Wang A, 2020. Competing protein–RNA interaction networks control multiphase intracellular organization. Cell 181: 306–324.e28. 10.1016/j.cell.2020.03.050 32302570 PMC7816278

[GAD350518PUTC192] Sato K, Sakai M, Ishii A, Maehata K, Takada Y, Yasuda K, Kotani T. 2022. Identification of embryonic RNA granules that act as sites of mRNA translation after changing their physical properties. iScience 25: 104344. 10.1016/j.isci.2022.104344 35620421 PMC9127168

[GAD350518PUTC193] Sauer M, Juranek SA, Marks J, De Magis A, Kazemier HG, Hilbig D, Benhalevy D, Wang X, Hafner M, Paeschke K. 2019. DHX36 prevents the accumulation of translationally inactive mRNAs with G4-structures in untranslated regions. Nat Commun 10: 2421. 10.1038/s41467-019-10432-5 31160600 PMC6547686

[GAD350518PUTC194] Schmidt H, Putnam A, Rasoloson D, Seydoux G. 2021. Protein-based condensation mechanisms drive the assembly of RNA-rich P granules. Elife 10: e63698. 10.7554/eLife.63698 34106046 PMC8238508

[GAD350518PUTC195] Schütz S, Nöldeke ER, Sprangers R. 2017. A synergistic network of interactions promotes the formation of in vitro processing bodies and protects mRNA against decapping. Nucleic Acids Res 45: 6911–6922. 10.1093/nar/gkx353 28472520 PMC5499654

[GAD350518PUTC196] Seim I, Posey AE, Snead WT, Stormo BM, Klotsa D, Pappu RV, Gladfelter AS. 2022. Dilute phase oligomerization can oppose phase separation and modulate material properties of a ribonucleoprotein condensate. Proc Natl Acad Sci 119: e2120799119. 10.1073/pnas.2120799119 35333653 PMC9060498

[GAD350518PUTC197] Sepulveda G, Antkowiak M, Brust-Mascher I, Mahe K, Ou T, Castro NM, Christensen LN, Cheung L, Jiang X, Yoon D, 2018. Co-translational protein targeting facilitates centrosomal recruitment of PCNT during centrosome maturation in vertebrates. Elife 7: e34959. 10.7554/eLife.34959 29708497 PMC5976437

[GAD350518PUTC198] Sheth U, Parker R. 2003. Decapping and decay of messenger RNA occur in cytoplasmic processing bodies. Science 300: 805–808. 10.1126/science.1082320 12730603 PMC1876714

[GAD350518PUTC199] Sheth U, Pitt J, Dennis S, Priess JR. 2010. Perinuclear P granules are the principal sites of mRNA export in adult *C. elegans* germ cells. Development 137: 1305–1314. 10.1242/dev.044255 20223759 PMC2847466

[GAD350518PUTC200] Sheu-Gruttadauria J, MacRae IJ. 2018. Phase transitions in the assembly and function of human miRISC. Cell 173: 946–957.e16. 10.1016/j.cell.2018.02.051 29576456 PMC5935535

[GAD350518PUTC201] Shin Y, Brangwynne CP. 2017. Liquid phase condensation in cell physiology and disease. Science 357: eaaf4382. 10.1126/science.aaf4382 28935776

[GAD350518PUTC202] Shukla A, Yan J, Pagano DJ, Dodson AE, Fei Y, Gorham J, Seidman JG, Wickens M, Kennedy S. 2020. Poly(UG)-tailed RNAs in genome protection and epigenetic inheritance. Nature 582: 283–288. 10.1038/s41586-020-2323-8 32499657 PMC8396162

[GAD350518PUTC203] Smith MR, Costa G. 2022. RNA-binding proteins and translation control in angiogenesis. FEBS J 289: 7788–7809. 10.1111/febs.16286 34796614

[GAD350518PUTC204] Smith J, Calidas D, Schmidt H, Lu T, Rasoloson D, Seydoux G. 2016. Spatial patterning of P granules by RNA-induced phase separation of the intrinsically-disordered protein MEG-3. Elife 5: e21337. 10.7554/eLife.21337 27914198 PMC5262379

[GAD350518PUTC205] So C, Cheng S, Schuh M. 2021. Phase separation during germline development. Trends Cell Biol 31: 254–268. 10.1016/j.tcb.2020.12.004 33455855

[GAD350518PUTC206] Spaulding EL, Feidler AM, Cook LA, Updike DL. 2022. RG/RGG repeats in the *C. elegans* homologs of nucleolin and GAR1 contribute to sub-nucleolar phase separation. Nat Commun 13: 6585. 10.1038/s41467-022-34225-5 36329008 PMC9633708

[GAD350518PUTC207] Standart N, Weil D. 2018. P-bodies: cytosolic droplets for coordinated mRNA storage. Trends Genet 34: 612–626. 10.1016/j.tig.2018.05.005 29908710

[GAD350518PUTC208] Starke EL, Zius K, Barbee SA. 2022. FXS causing missense mutations disrupt FMRP granule formation, dynamics, and function. PLoS Genet 18: e1010084. 10.1371/journal.pgen.1010084 35202393 PMC8903291

[GAD350518PUTC209] Stoecklin G, Mayo T, Anderson P. 2006. ARE-mRNA degradation requires the 5′–3′ decay pathway. EMBO Rep 7: 72–77. 10.1038/sj.embor.7400572 16299471 PMC1369226

[GAD350518PUTC210] Strulson CA, Molden RC, Keating CD, Bevilacqua PC. 2012. RNA catalysis through compartmentalization. Nature Chem 4: 941–946. 10.1038/nchem.1466 23089870

[GAD350518PUTC211] Strzelecka M, Trowitzsch S, Weber G, Lührmann R, Oates AC, Neugebauer KM. 2010. Coilin-dependent snRNP assembly is essential for zebrafish embryogenesis. Nat Struct Mol Biol 17: 403–409. 10.1038/nsmb.1783 20357773

[GAD350518PUTC212] Sundby AE, Molnar RI, Claycomb JM. 2021. Connecting the dots: linking *Caenorhabditis elegans* small RNA pathways and germ granules. Trends Cell Biol 31: 387–401. 10.1016/j.tcb.2020.12.012 33526340

[GAD350518PUTC213] Tartakoff A, DiMario P, Hurt E, McStay B, Panse VG, Tollervey D. 2022. The dual nature of the nucleolus. Genes Dev 36: 765–769. 10.1101/gad.349748.122 36342833 PMC9480854

[GAD350518PUTC214] Tauber D, Tauber G, Khong A, Van Treeck B, Pelletier J, Parker R. 2020. Modulation of RNA condensation by the DEAD-box protein eIF4A. Cell 180: 411–426.e16. 10.1016/j.cell.2019.12.031 31928844 PMC7194247

[GAD350518PUTC215] Teixeira D, Sheth U, Valencia-Sanchez MA, Brengues M, Parker R. 2005. Processing bodies require RNA for assembly and contain nontranslating mRNAs. RNA 11: 371–382. 10.1261/rna.7258505 15703442 PMC1370727

[GAD350518PUTC216] Tharun S, Parker R. 2001. Targeting an mRNA for decapping: displacement of translation factors and association of the Lsm1p–7p complex on deadenylated yeast mRNAs. Mol Cell 8: 1075–1083. 10.1016/S1097-2765(01)00395-1 11741542

[GAD350518PUTC217] Thiry M, Lafontaine DLJ. 2005. Birth of a nucleolus: the evolution of nucleolar compartments. Trends Cell Biol 15: 194–199. 10.1016/j.tcb.2005.02.007 15817375

[GAD350518PUTC218] Thomas L, Ismail BT, Askjaer P, Seydoux G. 2022. Cytoplasmic nucleoporin foci are stress-sensitive, non-essential condensates in *C. elegans*. bioRxiv 10.1101/2022.08.22.504855

[GAD350518PUTC219] Thomas L, Putnam A, Folkmann A. 2023. Germ granules in development. Development 150: dev201037. 10.1242/dev.201037 36715566 PMC10165536

[GAD350518PUTC220] Tibble RW, Depaix A, Kowalska J, Jemielity J, Gross JD. 2021. Biomolecular condensates amplify mRNA decapping by biasing enzyme conformation. Nat Chem Biol 17: 615–623. 10.1038/s41589-021-00774-x 33767388 PMC8476181

[GAD350518PUTC221] Trcek T, Lehmann R. 2019. Germ granules in *Drosophila*. Traffic 20: 650–660. 10.1111/tra.12674 31218815 PMC6771631

[GAD350518PUTC222] Trcek T, Douglas TE, Grosch M, Yin Y, Eagle WVI, Gavis ER, Shroff H, Rothenberg E, Lehmann R. 2020. Sequence-independent self-assembly of germ granule mRNAs into homotypic clusters. Mol Cell 78: 941–950.e12. 10.1016/j.molcel.2020.05.008 32464092 PMC7325742

[GAD350518PUTC223] Trojanowski J, Frank L, Rademacher A, Mücke N, Grigaitis P, Rippe K. 2022. Transcription activation is enhanced by multivalent interactions independent of phase separation. Mol Cell 82: 1878–1893.e10. 10.1016/j.molcel.2022.04.017 35537448

[GAD350518PUTC224] Tsang B, Arsenault J, Vernon RM, Lin H, Sonenberg N, Wang L-Y, Bah A, Forman-Kay JD. 2019. Phosphoregulated FMRP phase separation models activity-dependent translation through bidirectional control of mRNA granule formation. Proc Natl Acad Sci 116: 4218–4227. 10.1073/pnas.1814385116 30765518 PMC6410804

[GAD350518PUTC225] Turner-Bridger B, Jakobs M, Muresan L, Wong HH-W, Franze K, Harris WA, Holt CE. 2018. Single-molecule analysis of endogenous β-actin mRNA trafficking reveals a mechanism for compartmentalized mRNA localization in axons. Proc Natl Acad Sci 115: E9697–E9706. 10.1073/pnas.1806189115 30254174 PMC6187124

[GAD350518PUTC227] Uebel CJ, Phillips CM. 2019. Phase-separated protein dynamics are affected by fluorescent tag choice. MicroPubl Biol 2019: 10.17912/micropub.biology.000143. 10.17912/micropub.biology.000143 PMC7241484 32440657

[GAD350518PUTC228] Uebel CJ, Anderson DC, Mandarino LM, Manage KI, Aynaszyan S, Phillips CM. 2018. Distinct regions of the intrinsically disordered protein MUT-16 mediate assembly of a small RNA amplification complex and promote phase separation of Mutator foci. PLoS Genet 14: e1007542. 10.1371/journal.pgen.1007542 30036386 PMC6072111

[GAD350518PUTC229] Van Treeck B, Protter DSW, Matheny T, Khong A, Link CD, Parker R. 2018. RNA self-assembly contributes to stress granule formation and defining the stress granule transcriptome. Proc Natl Acad Sci 115: 2734–2739. 10.1073/pnas.1800038115 29483269 PMC5856561

[GAD350518PUTC230] Vidya E, Duchaine TF. 2022. Eukaryotic mRNA decapping activation. Front Genet 13: 832547. 10.3389/fgene.2022.832547 35401681 PMC8984151

[GAD350518PUTC231] Wallace EWJ, Kear-Scott JL, Pilipenko EV, Schwartz MH, Laskowski PR, Rojek AE, Katanski CD, Riback JA, Dion MF, Franks AM, 2015. Reversible, specific, active aggregates of endogenous proteins assemble upon heat stress. Cell 162: 1286–1298. 10.1016/j.cell.2015.08.041 26359986 PMC4567705

[GAD350518PUTC232] Wan G, Fields BD, Spracklin G, Shukla A, Phillips CM, Kennedy S. 2018. Spatiotemporal regulation of liquid-like condensates in epigenetic inheritance. Nature 557: 679–683. 10.1038/s41586-018-0132-0 29769721 PMC6479227

[GAD350518PUTC233] Wang JT, Smith J, Chen B-C, Schmidt H, Rasoloson D, Paix A, Lambrus BG, Calidas D, Betzig E, Seydoux G. 2014. Regulation of RNA granule dynamics by phosphorylation of serine-rich, intrinsically disordered proteins in *C. elegans*. Elife 3: e04591. 10.7554/eLife.04591 25535836 PMC4296509

[GAD350518PUTC234] Wang C, Schmich F, Srivatsa S, Weidner J, Beerenwinkel N, Spang A. 2018. Context-dependent deposition and regulation of mRNAs in P-bodies. Elife 7: e29815. 10.7554/eLife.29815 29297464 PMC5752201

[GAD350518PUTC235] Wei M-T, Chang Y-C, Shimobayashi SF, Shin Y, Strom AR, Brangwynne CP. 2020. Nucleated transcriptional condensates amplify gene expression. Nat Cell Biol 22: 1187–1196. 10.1038/s41556-020-00578-6 32929202

[GAD350518PUTC236] Westerich KJ, Tarbashevich K, Gupta A, 2022. Patterning of phase-separated condensates by Dnd1 controls cell fate. bioRxiv 10.1101/2022.10.20.512863

[GAD350518PUTC237] Wilbertz JH, Voigt F, Horvathova I, Roth G, Zhan Y, Chao JA. 2019. Single-molecule imaging of mRNA localization and regulation during the integrated stress response. Mol Cell 73: 946–958.e7. 10.1016/j.molcel.2018.12.006 30661979

[GAD350518PUTC238] Wilczynska A, Aigueperse C, Kress M, Dautry F, Weil D. 2005. The translational regulator CPEB1 provides a link between dcp1 bodies and stress granules. J Cell Sci 118: 981–992. 10.1242/jcs.01692 15731006

[GAD350518PUTC239] Xie SQ, Martin S, Guillot PV, Bentley DL, Pombo A. 2006. Splicing speckles are not reservoirs of RNA polymerase II, but contain an inactive form, phosphorylated on serine2 residues of the C-terminal domain. Mol Biol Cell 17: 1723–1733. 10.1091/mbc.E05-08-0726 16467386 PMC1415300

[GAD350518PUTC240] Xing W, Muhlrad D, Parker R, Rosen MK. 2020. A quantitative inventory of yeast P body proteins reveals principles of composition and specificity. Elife 9: e56525. 10.7554/eLife.56525 32553117 PMC7373430

[GAD350518PUTC241] Yang W-H, Yu JH, Gulick T, Bloch KD, Bloch DB. 2006. RNA-associated protein 55 (RAP55) localizes to mRNA processing bodies and stress granules. RNA 12: 547–554. 10.1261/rna.2302706 16484376 PMC1421083

[GAD350518PUTC242] Yang P, Mathieu C, Kolaitis RM, Zhang P, Messing J, Yurtsever U, Yang Z, Wu J, Li Y, Pan Q, 2020. G3BP1 is a tunable switch that triggers phase separation to assemble stress granules. Cell 181: 325–345.e28. 10.1016/j.cell.2020.03.046 32302571 PMC7448383

[GAD350518PUTC243] Yao R-W, Xu G, Wang Y, Shan L, Luan P-F, Wang Y, Wu M, Yang L-Z, Xing Y-H, Yang L, 2019. Nascent pre-rRNA sorting via phase separation drives the assembly of dense fibrillar components in the human nucleolus. Mol Cell 76: 767–783.e11. 10.1016/j.molcel.2019.08.014 31540874

[GAD350518PUTC244] Yoo H, Bard JAM, Pilipenko EV, Drummond DA. 2022. Chaperones directly and efficiently disperse stress-triggered biomolecular condensates. Mol Cell 82: 741–755.e11. 10.1016/j.molcel.2022.01.005 35148816 PMC8857057

[GAD350518PUTC245] Youn J-Y, Dunham WH, Hong SJ, Knight JDR, Bashkurov M, Chen GI, Bagci H, Rathod B, MacLeod G, Eng SWM, 2018. High-density proximity mapping reveals the subcellular organization of mRNA-associated granules and bodies. Mol Cell 69: 517–532.e11. 10.1016/j.molcel.2017.12.020 29395067

[GAD350518PUTC246] Zhang H, Elbaum-Garfinkle S, Langdon EM, Taylor N, Occhipinti P, Bridges AA, Brangwynne CP, Gladfelter AS. 2015. RNA controls PolyQ protein phase transitions. Mol Cell 60: 220–230. 10.1016/j.molcel.2015.09.017 26474065 PMC5221516

[GAD350518PUTC247] Zhang L, Zhang Y, Chen Y, Gholamalamdari O, Wang Y, Ma J, Belmont AS. 2021. TSA-seq reveals a largely conserved genome organization relative to nuclear speckles with small position changes tightly correlated with gene expression changes. Genome Res 31: 251–264. 10.1101/gr.266239.120 PMC7849416 33355299

[GAD350518PUTC249] Zhao H, Wu D, Nguyen A, Li Y, Adão RC, Valkov E, Patterson GH, Piszczek G, Schuck P. 2021. Energetic and structural features of SARS-CoV-2 N-protein co-assemblies with nucleic acids. iScience 24: 102523. 10.1016/j.isci.2021.102523 33997662 PMC8103780

